# Dinitrogen Binding at a Trititanium Chloride Complex and Its Conversion to Ammonia under Ambient Conditions

**DOI:** 10.1002/anie.202204544

**Published:** 2022-07-11

**Authors:** Estefanía del Horno, Jesús Jover, Miguel Mena, Adrián Pérez‐Redondo, Carlos Yélamos

**Affiliations:** ^1^ Departamento de Química Orgánica y Química Inorgánica Instituto de Investigación Química “Andrés M. del Río” (IQAR) Universidad de Alcalá 28805 Alcalá de Henares-Madrid Spain; ^2^ Secció de Química Inorgànica Departament de Química Inorgànica i Orgànica Institut de Química Teòrica i Computacional (IQTC-UB) Universitat de Barcelona Martí i Franquès 1-11 08028 Barcelona Spain

**Keywords:** Cluster Compounds, Density Functional Calculations, Nitrogen Fixation, Reduction, Titanium

## Abstract

Reaction of [TiCp*Cl_3_] (Cp*=η^5^‐C_5_Me_5_) with one equivalent of magnesium in tetrahydrofuran at room temperature affords the paramagnetic trinuclear complex [{TiCp*(μ‐Cl)}_3_(μ_3_‐Cl)], which reacts with dinitrogen under ambient conditions to give the diamagnetic derivative [{TiCp*(μ‐Cl)}_3_(μ_3_‐η^1^ : η^2^ : η^2^‐N_2_)] and the titanium(III) dimer [{TiCp*Cl(μ‐Cl)}_2_]. The structure of the trinuclear mixed‐valence complexes has been studied by experimental and theoretical methods and the latter compound represents the first well‐defined example of the μ_3_‐η^1^ : η^2^ : η^2^ coordination mode of the dinitrogen molecule. The reaction of [{TiCp*(μ‐Cl)}_3_(μ_3_‐η^1^ : η^2^ : η^2^‐N_2_)] with excess HCl in tetrahydrofuran results in clean NH_4_Cl formation with regeneration of the starting material [TiCp*Cl_3_]. Therefore, a cyclic ammonia synthesis under ambient conditions can be envisioned by alternating N_2_/HCl atmospheres in a [TiCp*Cl_3_]/Mg(excess) reaction mixture in tetrahydrofuran.

## Graphical abstract

The reduction of [TiCp*Cl_3_] with magnesium leads to a mixed‐valence titanium species capable of reacting with dinitrogen to form a stable trinuclear complex with a η^1^ : η^2^ : η^2^‐N_2_ ligand. This dinitrogen complex further reacts cleanly with HCl to produce NH_4_Cl with regeneration of the titanium precursor. Thus, a cyclic ammonia synthesis under ambient conditions can be envisaged.

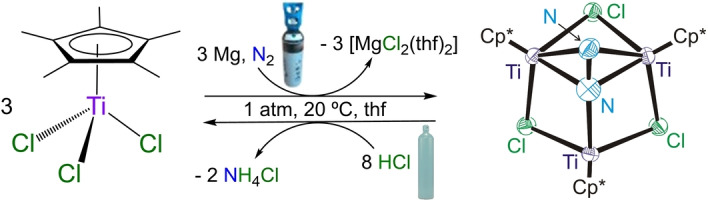

## Introduction

The synthesis of ammonia constitutes one of the most important chemical processes at the industrial level as illustrated by the production of about 150 million metric tons of this molecule in 2021.^[1]^ Most of this amount is used to supply nitrogenous fertilizers for world‐wide food production, but a smaller part is dedicated to several large‐scale synthesis of derivatives in the explosives, plastics, and textiles industries, among others. While it is expected a further increase in the ammonia demand for these current uses due to the human population growth, ammonia is also gaining interest as the energy carrier of the future.^[2]^ Today, the artificial synthesis of ammonia relies on the Haber‐Bosch process, developed over 100 years ago, and performed in large‐scale plants where dinitrogen and dihydrogen gases are combined under harsh conditions (300–500 °C and 200–300 bar) in the presence of a heterogeneous catalyst.^[3]^ However, this extremely energy‐intensive industrial process consumes about 2 % of the world′s total energy and generates ≈1 % of global CO_2_ emissions annually and, unsurprisingly, the development of a sustainable production of ammonia has been identified as one of the IUPAC Top Ten Emerging Technologies in Chemistry 2021.^[4]^

Inspired in the natural nitrogen fixation carried out under ambient conditions by certain microorganisms which convert N_2_ into ammonia using electrons and protons at nitrogenase enzymes,^[5]^ chemists have long explored biological‐models and homogeneous catalysts capable of operating at milder conditions.^[6]^ In particular, inorganic and organometallic researchers have intensively investigated the coordination and activation of the dinitrogen molecule at one or multiple metal atoms in well‐defined complexes.^[7]^ As a result, various catalytic homogeneous systems for conversion of N_2_ to NH_3_ under mild conditions have been developed in the last two decades.[^8^, ^9^] The methods in those catalytic systems involve the addition of a high excess of external reducing agents and protic acids to solutions of well‐defined transition metal complexes in a dinitrogen atmosphere. While molybdenum and iron complexes have received traditionally most attention in the field since they are naturally present in nitrogenase enzymes,^[8]^ catalytic systems using other metals have been reported in the last years (e.g., Co, Ru, Os, V, and Ti).^[9a]^ For instance, Liddle and co‐workers have recently reported triamidoamine‐ligated titanium dinitrogen complexes competent for catalytic ammonia formation,^[10]^ and Okuda and co‐workers used similar compounds for the catalytic conversion of N_2_ to N(SiMe_3_)_3_.^[11]^

The fixation of dinitrogen by titanium species has been known for decades,^[12]^ and nowadays a variety of coordination modes of the N_2_ unit to the titanium centers is well‐documented.^[7]^ In addition to a few examples with end‐on (η^1^) binding mode,^[13]^ literature reveals multiple references of structurally characterized end‐on/end‐on (μ‐η^1^ : η^1^),^[14]^ side‐on/side‐on (μ‐η^2^ : η^2^),[^13c^, ^15^] and end‐on/side‐on (μ‐η^1^ : η^2^)^[16]^ bridging dinitrogen in dinuclear complexes, and even the rare end‐on/end‐on/side‐on (μ_3_‐η^1^ : η^1^ : η^2^) coordination mode at three titanium centers has been reported.[^15b^, ^17^] Moreover, the end‐on/side‐on/side‐on (μ_3_‐η^1^ : η^2^ : η^2^) coordination mode of dinitrogen is only known for titanium, and was characterized by NMR spectroscopy at low temperature (−30 °C) in the intermediate [{Ti(η^5^‐C_5_Me_4_SiMe_3_)(μ‐H)}_3_(μ_3_‐η^1^ : η^2^ : η^2^‐N_2_)] before the cleavage of the N−N bond at higher temperatures (−10 °C) took place.^[18]^ Indeed, a recent theoretical study on the dissociative adsorption of N_2_ on trinuclear carbide cluster anions V_3_C_4_^−^ identified a key intermediate with a μ_3_‐η^1^ : η^2^ : η^2^‐N_2_ fragment that enables the facile cleavage of the N≡N bond.^[19]^

**Scheme 1:**
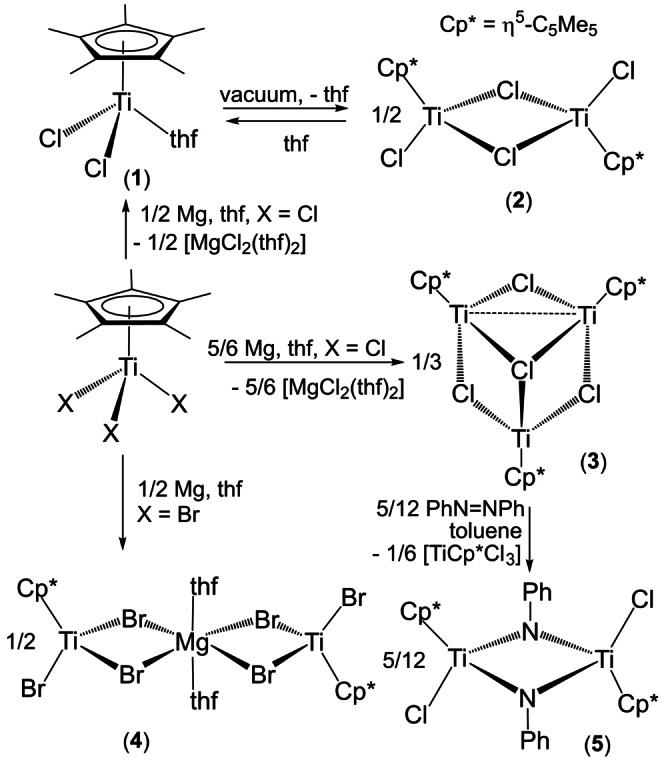
Reductions of [TiCp*X_3_] with magnesium under argon atmosphere.

As part of our recent research program on low‐valent titanium complexes,^[20]^ we have reported the structure and properties of several half‐sandwich titanium(III) derivatives [TiCp*Cl_2_] (Cp*=η^5^‐C_5_Me_5_) prepared by thermal decomposition or hydrogenolysis of [TiCp*Cl_2_Me] or by treatment of [TiCp*Cl_3_] with appropriated ratios of conventional reductants (Li_3_N, LiAlH_4_, Mg).[^21^, ^22^] For instance, the reduction of [TiCp*Cl_3_] with a half equivalent of magnesium in tetrahydrofuran afforded a blue solution of [TiCp*Cl_2_(thf)] (**1**), which under vacuum led to the dinuclear compound [{TiCp*Cl(μ‐Cl)}_2_] (**2**) (Scheme 1). While none of these titanium(III) species reacted with dinitrogen, here we report that further reduction with magnesium affords a mixed‐valence trinuclear complex [{TiCp*(μ‐Cl)}_3_(μ_3_‐Cl)] (**3**) capable of incorporating N_2_ under ambient conditions in solution to give a stable derivative with a μ_3_‐η^1^ : η^2^ : η^2^‐N_2_ ligand. This dinitrogen complex reacts with excess HCl to regenerate [TiCp*Cl_3_] with NH_4_Cl formation, and several cycles alternating N_2_/HCl atmospheres on a [TiCp*Cl_3_]/Mg(excess)/thf system can be performed to produce NH_4_Cl on a relatively large scale.

## Results and Discussion

The treatment of [TiCp*Cl_3_] with one equivalent of magnesium in tetrahydrofuran at room temperature gave the mixed‐valence trinuclear complex [{TiCp*(μ‐Cl)}_3_(μ_3_‐Cl)] (**3**) (Scheme 1). In view of the stoichiometry of the reaction, Mg metal is in a slight excess but this is easily removed, along with the [MgCl_2_(thf)_2_] by‐product, after extraction with toluene to afford **3** as a brown solid in 87 % yield. The ^1^H‐NMR spectra of **3** in [D_6_]benzene or [D_8_]tetrahydrofuran solutions revealed one broad resonance at *δ*=11.0–10.9 ppm (Δν_1/2_=50 Hz) for the η^5^‐C_5_Me_5_ ligands. The paramagnetic nature of **3** was confirmed by an Evans method determination of its magnetic susceptibility (μ_eff_=2.17 μ_B_, 293 K, C_6_D_6_ solution), which is only slightly higher than that expected for one unpaired electron in the complex.^[23]^ Analogous reaction of [TiCp*Br_3_] with magnesium (0.5 or 1 equiv) in tetrahydrofuran gave the titanium(III)‐magnesium species [{TiCp*Br(μ‐Br)_2_}_2_Mg(thf)_2_] (**4**) as a brown‐green solid in up to 77 % yield. The ^1^H‐NMR spectrum of **4** in [D_6_]benzene is silent and its effective magnetic moment in solution at room temperature is 2.25 μ_B_. The X‐ray crystal structure of **4** shows two {TiCp*Br} units connected by two μ‐Br ligands to a magnesium atom which completes a distorted octahedral geometry with two additional tetrahydrofuran ligands in a *trans* disposition (Figure S1 of the Supporting Information),^[24]^ similar to that found in [{Ti(η^5^‐C_5_H_5_)_2_(μ‐Cl)_2_}_2_Mg(thf)_2_].^[25]^

**Figure 1:**
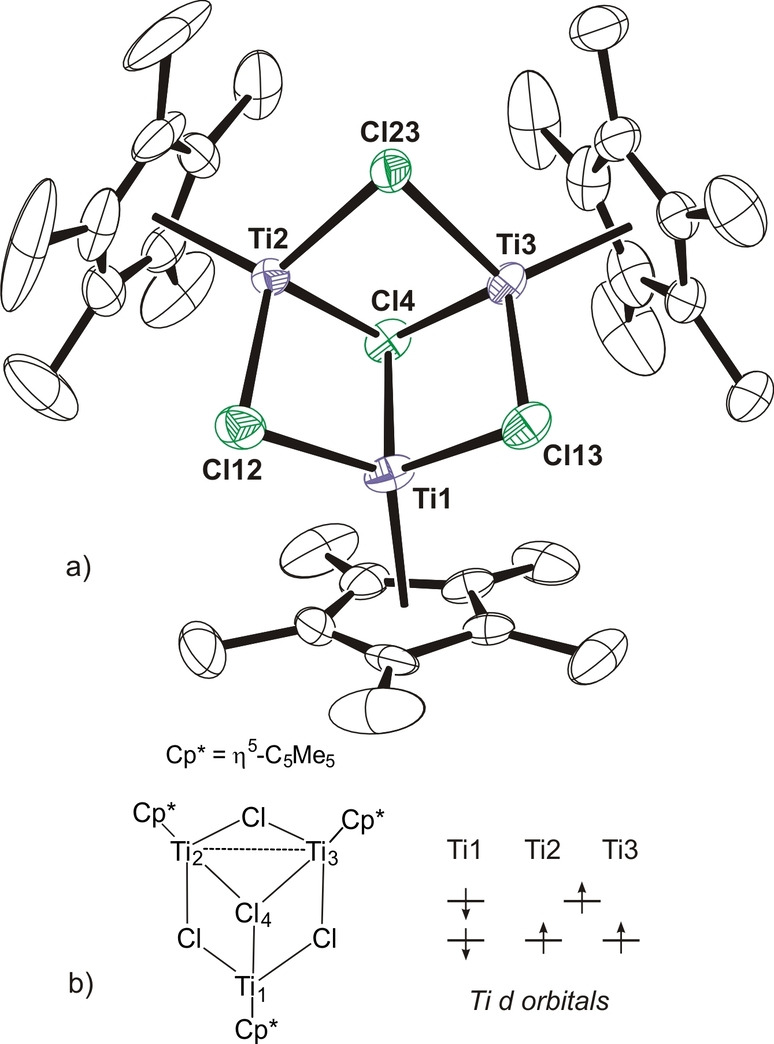
a) Perspective view of complex **3** (thermal ellipsoids at the 50 % probability level). Hydrogen atoms of the η^5^‐C_5_Me_5_ ligands are omitted for clarity. Selected lengths [Å] and angles [°]: Ti−Ti 2.872(1)–3.033(1), Ti−μCl av. 2.383(7), Ti−Cl(4) 2.399(1)–2.423(1), Cl−Ti−Cl 95.7(1)–101.2(1), Cl(4)−Ti−Cl 99.0(1)–106.3(1), Ti−Cl−Ti 74.5(1)–78.8(1), Ti−Cl(4)−Ti 73.0(1)–78.3(1). b) Most stable computed doublet electronic structure for compound **3**.

The crystal structure of **3** shows a nearly equilateral triangle of titanium atoms with Ti−Ti separations in the range of 2.872(1)–3.033(1) Å (Figure 1a). Each edge of the triangle is bridged by one chloride ligand and one face is capped by a fourth chloride. Therefore, each titanium is bonded to one η^5^‐C_5_Me_5_ group and three chloride ligands to give a typical three‐legged piano‐stool geometry. Given the total negative charges (7−) of the η^5^‐C_5_Me_5_ and μ_n_‐Cl ligands and the nearly *C*_3v_ symmetry of complex **3**, a mixed‐valence Ti^II^/Ti^III^ complex with an average oxidation state of +2.33 for each metal could be anticipated.

Nevertheless, the electronic structure was studied by density functional theory (DFT) calculations and several complexes with different electronic structures containing one Ti^III^ and two Ti^II^ centers were computed. As for the spin state of the calculations, different doublet structures have been calculated with varied initial electronic distributions. Some quartet structures have been also computed, but these were discarded because of their relatively high energies. The electronic structure for the most stable doublet is depicted in Figure 1b.

**Figure 2:**
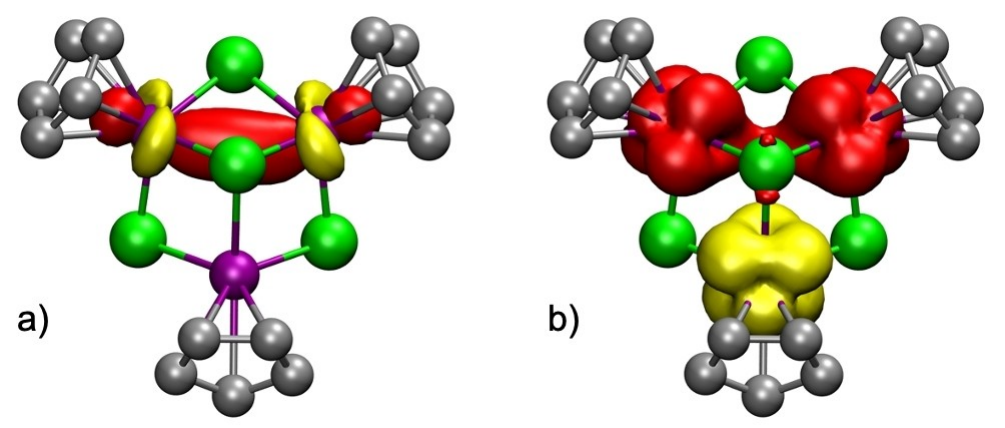
Computed a) half full Ti2−Ti3 alpha bonding orbital and b) spin density for compound **3**. Methyl groups of the η^5^‐C_5_Me_5_ ligands are omitted for clarity.

The electronic structure extracted from the computed NBO orbitals confirms that the complex should be considered a mixed‐valence compound. While the Ti1 atom has two unpaired electrons in beta d‐orbitals and should be considered a Ti^II^ center, the other two titanium atoms have each one unpaired electron on alpha d‐orbitals (Figure S3 of the Supporting Information). In addition, Ti2 and Ti3 atoms share an extra electron in a Ti−Ti alpha bonding orbital (Figure 2a) and should have a formal +2.5 oxidation state. The computed Ti−Ti bond order of 0.26 is slightly lower than the expected 0.50 value but it indicates a certain bond order between the titanium atoms. The overall spin density (Figure 2b) for complex **3** results in one unpaired electron.

The mass spectrum (EI, 70 eV) of **3** shows the expected molecular ion for the trinuclear structure in the gas phase, although the base peak of the spectrum corresponds to the [TiCp*Cl] fragment. While compound **3** appears to be stable in [D_6_]benzene solution even at high temperatures according to ^1^H‐NMR spectroscopy, spectra taken of solutions of **3** in [D_8_]tetrahydrofuran after 48 h at room temperature showed the broad resonance of the tetrahydrofuran adduct **1** (ca. 10 % conversion) at *δ*=14.1 ppm (Δν_1/2_=186 Hz).^[21]^ Indeed the reactivity of **3** in [D_6_]benzene towards a variety of unsaturated substrates suggests a behavior as a source of titanium(II) and titanium(III) fragments (Scheme S1). Thus, reaction of **3** with bis(trimethylsilyl)acetylene at room temperature slowly gave a mixture of compound **2** and the previously described complex [{TiCp*{η^2^‐C_2_(SiMe_3_)_2_}(μ‐Cl)}_2_].^[26]^ Similarly, treatment of a solution of **3** with *N*‐(4‐methylbenzylidene)aniline led to the formation of **2** and the monomeric diamagnetic diazatitanacyclopentane [TiCp*Cl{N(Ph)CH(*p*‐tolyl)}_2_] reported by Beckhaus and co‐workers.^[27]^ Furthermore, addition of azobenzene to a solution of **3** generates a mixture of the imido‐bridged dinuclear complex [{TiCp*Cl(μ‐NPh)}_2_] (**5**) and [TiCp*Cl_3_] (Scheme 1). The latter compound results from the reaction at room temperature of the titanium(III) complex **2** with PhN=NPh as showed by an independent experiment in [D_6_]benzene which cleanly gave **5** and [TiCp*Cl_3_].^[28]^ The hitherto unknown brown complex **5** was isolated in a poor 48 % yield due to its similar solubility to that of [TiCp*Cl_3_], and was characterized by analytical and spectroscopic methods, as well as by an X‐ray crystal structure determination (Figure S2). The structural characterization of **5** reveals typical parameters of imido‐bridged titanium complexes.^[29]^

Analogous four‐electron reductive cleavage of the N=N double bond of azobenzene by low‐valent titanium species is well documented in the literature,[^28^, ^29^, ^30^] and we decided to explore the possibility of reducing the N≡N bond of molecular nitrogen. Thus, exposure of solutions of the paramagnetic complex **3** to 1 atm of dinitrogen at room temperature resulted in formation of one diamagnetic species according to ^1^H‐NMR spectroscopy. Spectra of a [D_6_]benzene solution taken after 5 days revealed the complete consumption of **3** and the appearance of two sharp singlets in a 2 : 1 ratio at *δ*=1.87 and 1.70 ppm attributable to the η^5^‐C_5_Me_5_ ligands of a diamagnetic molecule along with resonances due to the titanium(III) dimer **2**.^[21]^ The reaction of **3** in [D_8_]tetrahydrofuran with N_2_ at room temperature is completed after 24 h and the spectra showed only two singlets in a 2 : 1 ratio (*δ*=1.81 and 1.76 ppm) along with the broad resonance characteristic of complex **1**.

**Scheme 2:**
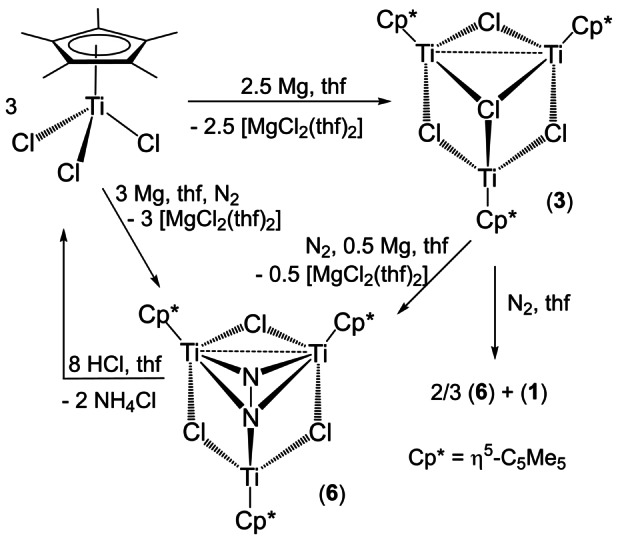
Reaction of [TiCp*Cl_3_] with magnesium under N_2_ atmosphere.

Indeed, exposure of a tetrahydrofuran solution of complex **3**, generated in situ by reaction of [TiCp*Cl_3_] with Mg (1 equiv) under argon, to N_2_ (1 atm) at room temperature for 16 h gave the trinuclear derivative [{TiCp*(μ‐Cl)}_3_(μ_3_‐η^1^ : η^2^ : η^2^‐N_2_)] (**6**) in 87 % yield (Scheme 2). The ^1^H‐NMR spectrum of the isolated brown solid of **6** does not show any resonance signal attributable to complexes **1** or **2**, since these titanium(III) species were further reduced with the slight excess of Mg used in the preparation of **3**. The analogous treatment of generated **3** in tetrahydrofuran solution with isotopically enriched ^15^N_2_ afforded **6‐^15^N** in 72 % isolated yield. Remarkably, the direct reduction of [TiCp*Cl_3_] with Mg under dinitrogen atmosphere also gave **6** as the major product but it is accompanied by minor amounts of paramagnetic species according to subsequent ^1^H‐NMR analysis. Presumably, this is due to partial reaction of generated **6** with magnesium, as demonstrated by an independent treatment of isolated **6** with excess Mg that led to unidentified paramagnetic products.

**Figure 3:**
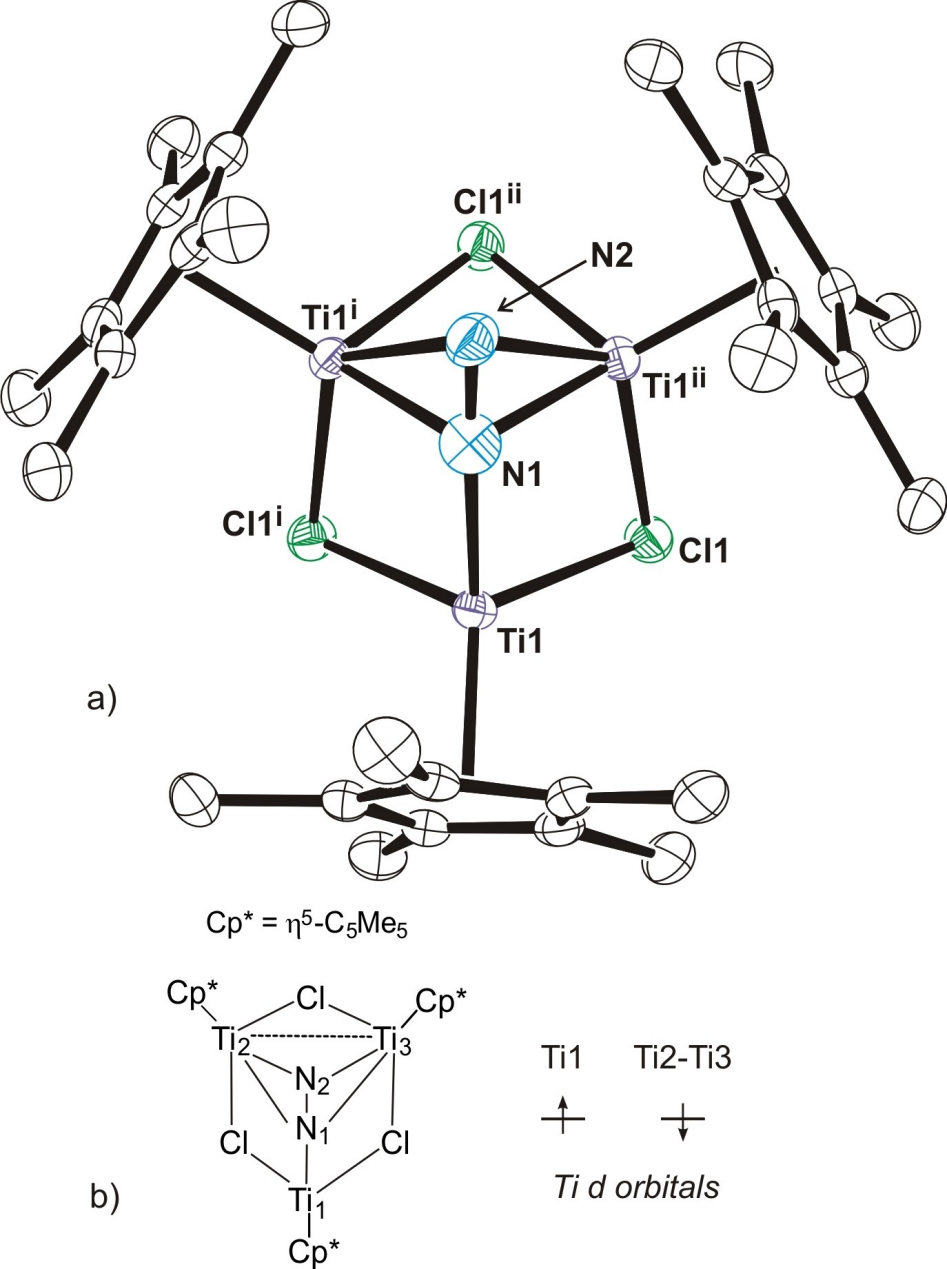
a) Perspective view of complex **6** (thermal ellipsoids at the 50 % probability level). Hydrogen atoms of the η^5^‐C_5_Me_5_ ligands are omitted for clarity. Selected lengths [Å] and angles [°]: Ti−Ti 2.936(1), Ti−Cl av. 2.395(4), Ti(1)−N(1) 2.017(3), Ti(1)^i^−N(2) 2.146(9), Ti(1)^ii^−N(2) 2.099(10), N(1)−N(2) 1.099(9), Cl−Ti−Cl 95.9(1), N(1)−Ti−Cl av. 95.5(1), Ti−Cl−Ti 75.6(1), Ti−N(1)−Ti 93.4(2), Ti(1)−N(1)−N(2) 170.2(7), Ti−N(2)−Ti 87.5(4). Symmetry code: (i) 1−*y*, *x*−*y*, *z*; (ii) 1−*x*+*y*, 1−*x*, *z*. b) Most stable computed singlet electronic structure for compound **6**.

Compound **6** exhibits a good solubility and stability at room temperature in tetrahydrofuran, toluene or benzene but it is poorly soluble in hexane. ^1^H‐ and ^13^C{^1^H}‐NMR spectra of **6** in [D_6_]benzene or [D_8_]tetrahydrofuran at room temperature showed resonance signals for two inequivalent η^5^‐C_5_Me_5_ ligands in accordance with a *C*_s_ symmetry in solution. The ^15^N‐NMR spectrum of **6‐^15^N** revealed two doublets at *δ*=95.4 and −0.4 ppm (^1^ *J*_N‐N_=19.8 Hz) referenced to MeNO_2_. A comparison of the IR spectra (KBr) of **6** and **6‐^15^N** allows the identification of the N−N stretching vibration (^14^N/^15^N at 1277/1234 cm^−1^) indicating a highly reduced N_2_ unit (cf. 1529 and 1076 cm^−1^ for the N−N IR or Raman stretching vibrations of *trans*‐N_2_H_2_ and N_2_H_4_, respectively).^[7d]^ Brown crystals of **6** for an X‐ray crystal structure determination were grown from a toluene/hexane solution at −35 °C. The molecular structure of **6** shows an equilateral triangle of titanium atoms with Ti−Ti separations of 2.936(1) Å (Figure 3a). Each edge of the triangle is bridged by one chloride ligand and one face is capped by a μ_3_‐η^1^ : η^2^ : η^2^‐N_2_ ligand. The structure exhibits a C_3_ axis that crosses through the N(1) atom, while N(2) is disordered in three equally occupied positions located on the three edges of the triangle. The N−N bond length of 1.099(9) Å is very similar to the 1.098(1) Å separation in free dinitrogen. However, the spectroscopic data of **6** (ν_NN_ frequency of 1277 cm^−1^ and ^15^N‐NMR resonances in the IR and ^15^N‐NMR spectra) are very different from those of free N_2_ (2330 cm^−1^ in Raman spectroscopy and δ=−70.4 ppm, respectively). The symmetry and disorder observed in the crystal structure determination of **6** probably explain the ambiguities about the geometric parameters of the [N_2_] unit.

Since the crystal structure did not allow an accurate determination of the dinitrogen ligand, the electronic structure of **6** was studied by DFT calculations. In spite of the diamagnetic character of this molecule, the charge assignment is complicated since the bridging N_2_ unit can adopt a range of different charges (i.e., [N_2_]^0^, [N_2_]^2−^, [N_2_]^4−^) and, depending on that, different spin states combined with different metal charge distributions had to be tested. The most stable computed singlet showed one alpha unpaired electron on Ti1 and a beta unpaired electron shared between Ti2 and Ti3, hence forming a Ti−Ti bond (Figure 3b). Again, the computed Ti−Ti bond order of 0.37 is slightly lower than the expected 0.50 value but it indicates a certain bond order between the titanium atoms. Thus, the Ti1 atom exhibits a +3 oxidation state while the Ti2 and Ti3 atoms have fractional oxidation states of +3.5.

**Figure 4:**
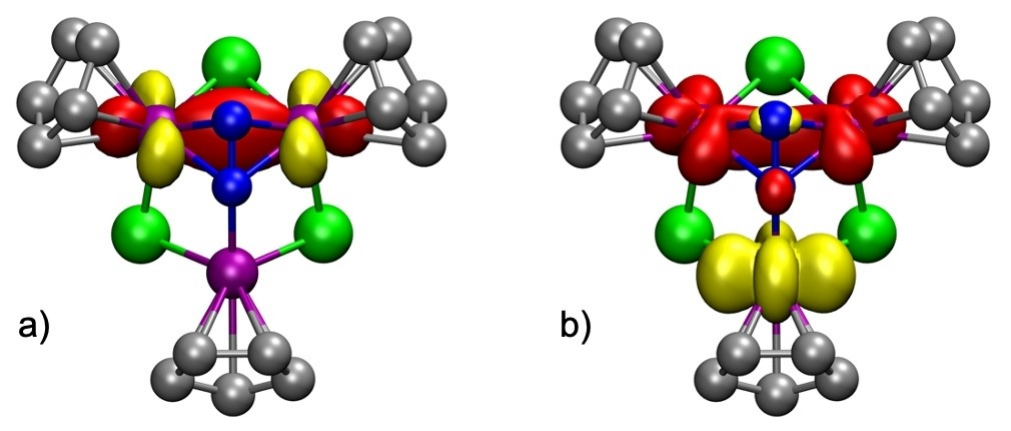
Computed a) half full Ti2−Ti3 beta bonding orbital and b) spin density for compound **6**. Methyl groups of the η^5^‐C_5_Me_5_ ligands are omitted for clarity.

The computed NBO orbitals for this mixed‐valence species show the alpha unpaired electron on Ti1 and the beta unpaired electron forming the Ti2−Ti3 bond (Figure 4a). In agreement with this metal‐metal bond, the computed Ti2−Ti3 distance is 2.84 Å while Ti1−Ti2 and Ti1−Ti3 are as long as 3.09 Å. This electronic arrangement produces the spin density picture shown in Figure 4b, where some spin delocalization in the N_2_ unit can be observed, although this is mainly due to spin polarization.

**Figure 5:**
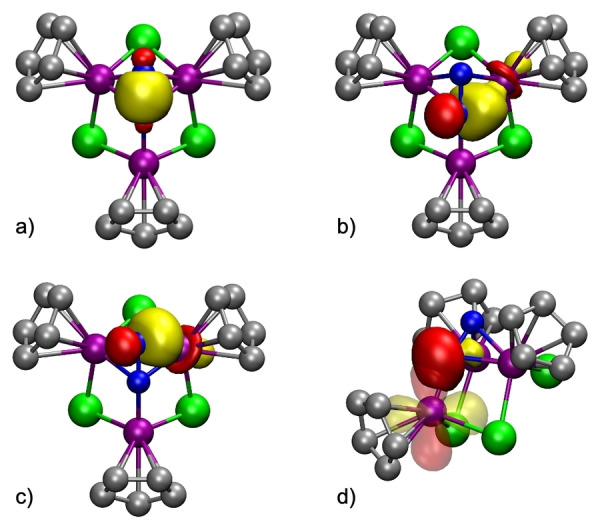
Compound **6** NBO orbitals within the N_2_ unit: a) N−N bond; b,c) Ti3−N1 and Ti3−N2 bonds (analogous NBO orbitals for Ti2−N1 and Ti2−N2 bonds were also found); d) Donor/acceptor interaction N1→Ti1 (solid and transparent orbitals represent electron donor (full) and acceptor (empty) orbitals, respectively). Methyl groups of the η^5^‐C_5_Me_5_ ligands are omitted for clarity.

The NBO analysis also shows that the N_2_ moiety has one N−N bond (Figure 5a) and one lone pair on each nitrogen atom (Figure S4), accounting each of them for two electrons. Additionally, both N1 and N2 have one single bond with Ti2 and Ti3 mainly with N character (ca. 75 %) (Figures 5b, c). All these data suggest that the dinitrogen unit should be considered an [N_2_]^4−^ ligand although the computed N−N distance of 1.31 Å is shorter than that found in hydrazine (1.45 Å) and closer to that (1.25 Å) of [N_2_]^2−^ in the diimine. Remarkably, the DFT computed N−N distance in complex **6** is very different to that (1.099(9) Å) found in the crystal structure, but the DFT value appears to be more accurate in this case according to the spectroscopic data as detailed below. The NBO analysis does not reveal any covalent bond between N1 and Ti1, probably because of the relative orientation of the NBO orbitals and the relative energy difference between both fragments. However, as shown in Figure 5d, there is a relevant donor/acceptor interaction N1→Ti1, which assists in stabilizing the structure. Indeed, the computed Ti1−N1 distance of 1.90 Å is the shortest of the titanium‐nitrogen bond lengths (e.g., Ti2−N1 and Ti2−N2 are 2.10 and 2.04 Å, respectively).

Since the computed structure of **6** cannot be directly compared to that determined by X‐ray crystallography, we have calculated the infrared and the ^15^N‐NMR spectra for this species. The computed N−N stretching band was found at 1233 cm^−1^ in good agreement with the experimental value of 1277 cm^−1^. Similarly, the computed ^15^N‐NMR resonances show a chemical shift difference of 93.1 ppm very similar to that found (Δ*δ*=95.8 ppm) between the doublets found in the experimental spectrum of **6** (95.4 and −0.4 ppm).

As mentioned above, the singular μ_3_‐η^1^ : η^2^ : η^2^ coordination mode of the dinitrogen ligand has been only described in the unstable intermediate [{Ti(η^5^‐C_5_Me_4_SiMe_3_)(μ‐H)}_3_(μ_3_‐η^1^ : η^2^ : η^2^‐N_2_)] characterized by NMR spectroscopy at low temperature.^[18]^ Since the oxidation states of the titanium atoms in this species were assigned formally, we have also computed the electronic structure of the analogous hydride complex [{TiCp*(μ‐H)}_3_(μ_3_‐η^1^ : η^2^ : η^2^‐N_2_)] (**6′**). The electronic structure of the most stable singlet species **6′** is the same of that described above for **6**, with one alpha unpaired electron on Ti1 and one beta unpaired electron shared between Ti2 and Ti3. The shape of the NBOs describing the Ti2−Ti3 bond, the unpaired electron on Ti1 and the spin density are pretty similar to those shown for compound **6** (Figure S5). In the same line, the NBOs representing the N1−N2 single bond, and the lone pairs on each N atom could be identified (Figure S6). In the calculated IR spectrum of **6′**, the N−N stretching band appeared at a lower wavenumber (1137 cm^−1^) than that computed for **6** (1233 cm^−1^). The calculated ^15^N‐NMR spectrum for **6′** revealed a Δ*δ* of 126.1 ppm between the two doublets of the μ_3_‐η^1^ : η^2^ : η^2^‐N_2_ ligand, which is slightly larger than that for complex **6** but smaller than that reported in the literature for the analogous [{Ti(η^5^‐C_5_Me_4_SiMe_3_)(μ‐H)}_3_(μ_3_‐η^1^ : η^2^ : η^2^‐N_2_)] (Δ*δ*=189.8 ppm).^[18]^

Monitoring by ^1^H and ^15^N‐NMR spectroscopy of a [D_6_]benzene solution of **6** or **6‐^15^N** revealed complete decomposition at temperatures higher than 40 °C to give unidentified paramagnetic species, but release of ^15^N_2_ was not detected in the ^15^N‐NMR spectra. The reactivity of **6** in [D_6_]benzene was explored by several NMR experiments at room temperature (Scheme S2). No apparent reaction between compound **6** and H_2_ (up to 5 atm), bis(trimethylsilyl)acetylene or chlorotrimethylsilane was observed. In contrast, the treatment of **6** with *N*‐(4‐methylbenzylidene)aniline and azobenzene cleanly afforded the previously described diazatitanacyclopentane [TiCp*Cl{N(Ph)CH(*p*‐tolyl)}_2_] and imido‐bridged [{TiCp*Cl(μ‐NPh)}_2_] (**5**) complexes, respectively. These reactions presumably occur by release of N_2_ as demonstrated by ^15^N‐NMR spectroscopy characterization of ^15^N_2_ (observed at *δ*=−70.4 ppm) in the reaction of **6‐^15^N** with 2,6‐dimethylphenylisocyanide. In the latter experiment, formation of the titanium(IV) derivative [(TiCp*Cl)_2_{μ‐N_2_C_2_(C_6_H_3_Me_2_)_2_}], reported by Teuben and co‐workers, was stablished by ^1^H‐ and ^13^C{^1^H}‐NMR spectroscopy.^[31]^ Interestingly, exposure of [D_6_]benzene solutions of **6** or **6‐^15^N** to anhydrous HCl (1 atm) immediately gave an orange suspension where, after removing the volatile components and dissolving the residue in [D_6_]DMSO, NH_4_Cl or ^15^NH_4_Cl were characterized by ^1^H‐NMR spectroscopy. Indeed, in a preparative scale reaction, exposure of a brown solution of complex **6** in tetrahydrofuran to HCl (1 atm) at room temperature resulted in the immediate formation of an orange suspension from which NH_4_Cl and [TiCp*Cl_3_] were isolated after work‐up as white (86 %) and red (75 %) powders, respectively (Scheme 2).

On the basis of the recyclability of the titanium complex [TiCp*Cl_3_], a catalytic synthesis of ammonia mediated by titanium can be envisaged (Equation 1). The system involves reduction of N_2_ with magnesium in thf solution and protonolysis with HCl in presence of a commercially available, or easily prepared in multigram quantities, titanium catalyst to give two equivalents of NH_4_Cl. The high solubility in tetrahydrofuran and poor solubility in toluene of the by‐product [MgCl_2_(thf)_2_] allow the easy separation of NH_4_Cl and [TiCp*Cl_3_] compounds in high purity. Thus, the treatment of a tetrahydrofuran solution of [TiCp*Cl_3_] (0.86 mmol) with an excess of Mg (30 equiv) under alternating N_2_ (30 min)/HCl (15 min) atmospheres for 9 cycles afforded NH_4_Cl (0.20 g, 3.75 mmol) in 72 % isolated yield (based on nine completed reactions of the titanium complex).

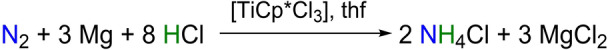




## Conclusion

In summary, we have shown that reduction of [TiCp*Cl_3_] with magnesium in tetrahydrofuran produces a trinuclear complex [{TiCp*(μ‐Cl)}_3_(μ_3_‐Cl)] with an average oxidation state of +2.33 for each metal. This mixed‐valence species behaves as a source of titanium(II) [TiCp*Cl] and titanium(III) [TiCp*Cl_2_] fragments according to its reactivity with unsaturated molecules. Thus, it reacts with dinitrogen under ambient conditions to give the diamagnetic derivative [{TiCp*(μ‐Cl)}_3_(μ_3_‐η^1^ : η^2^ : η^2^‐N_2_)] and the titanium(III) dihalide species [TiCp*Cl_2_] which is unreactive towards N_2_. According to experimental and theoretical studies, the dinitrogen complex can be described as a mixed‐valence compound with a [N_2_]^4−^ unit bonded in a side‐on fashion to two titanium atoms and through an important donor‐acceptor interaction between the lone pair of one nitrogen and an empty d‐orbital of the third titanium atom. This compound represents the first structurally and electronically characterized μ_3_‐η^1^ : η^2^ : η^2^ bound dinitrogen in a stable trimetallic complex. The dinitrogen ligand is highly activated and upon protonolysis with HCl produces NH_4_Cl in high yield with regeneration of the [TiCp*Cl_3_] precursor. Thus, a practical synthesis of ammonia mediated by a titanium complex can be envisioned via reduction of N_2_ with magnesium and protonation with HCl under ambient conditions.

## Conflict of interest

The authors declare the following competing financial interest: Universidad de Alcalá, E.d.H., A.P.‐R., M.M., and C.Y. have filed patents related to this work.

1

## Supporting information

As a service to our authors and readers, this journal provides supporting information supplied by the authors. Such materials are peer reviewed and may be re‐organized for online delivery, but are not copy‐edited or typeset. Technical support issues arising from supporting information (other than missing files) should be addressed to the authors.

Supporting Information

Supporting Information

Supporting Information

## Data Availability

The data that support the findings of this study are available in the supplementary material of this article.

## References

[1] Mineral Commodity Summaries 2022, U.S. Geological Survey, **2022**.

[2a] D. R. MacFarlane , P. V. Cherepanov , J. Choi , B. H. R. Suryanto , R. Y. Hodgetts , J. M. Bakker , F. M. Ferrero Vallana , A. N. Simonov , Joule 2020, 4, 1186–1205;

[2b] C. Smith , A. K. Hill , L. Torrente-Murciano , Energy Environ. Sci. 2020, 13, 331–344;

[2c] O. Elishav , B. Mosevitzky Lis , E. M. Miller , D. J. Arent , A. Valera-Medina , A. Grinberg Dana , G. E. Shter , G. S. Grader , Chem. Rev. 2020, 120, 5352–5436. 32501681 10.1021/acs.chemrev.9b00538

[3] H. Liu , Ammonia Synthesis Catalysts, Innovation and Practice, Chemical Industry Press and World Scientific, Singapore and Beijing, 2013.

[4] F. Gomollón-Bel , Chem. Int. 2021, 43, 13–20.

[5] L. C. Seefeldt , Z.-Y. Yang , D. A. Lukoyanov , D. F. Harris , D. R. Dean , S. Raugei , B. M. Hoffman , Chem. Rev. 2020, 120, 5082–5106. 32176472 10.1021/acs.chemrev.9b00556 PMC7703680

[6a] H.-P. Jia , E. A. Quadrelli , Chem. Soc. Rev. 2014, 43, 547–564; 24108246 10.1039/c3cs60206k

[6b] S. L. Foster , S. I. Perez Bakovic , R. D. Duda , S. Maheshwari , R. D. Milton , S. D. Minteer , M. J. Janik , J. N. Renner , L. F. Greenlee , Nat. Catal. 2018, 1, 490–500;

[6c] J. G. Chen , R. M. Crooks , L. C. Seefeldt , K. L. Bren , R. M. Bullock , M. Y. Darensbourg , P. L. Holland , B. Hoffman , M. J. Janik , A. K. Jones , M. G. Kanatzidis , P. King , K. M. Lancaster , S. V. Lymar , P. Pfromm , W. F. Schneider , R. R. Schrock , Science 2018, 360, eaar6611. 29798857 10.1126/science.aar6611 PMC6088796

[7] For selected recent reviews, see:

[7a] M. D. Walter , Adv. Organomet. Chem. 2016, 65, 261–377;

[7b] R. J. Burford , M. D. Fryzuk , Nat. Chem. Rev. 2017, 1, 0026;

[7c] Nitrogen Fixation in Top. Organomet. Chem., Vol. 60 (Ed.: Y. Nishibayashi ), Springer, Berlin, 2017;

[7d] Transition Metal-Dinitrogen Complexes (Ed.: Y. Nishibayashi ), Wiley-VCH, Weinheim, 2019;

[7e] D. Singh , W. R. Buratto , J. F. Torres , L. J. Murray , Chem. Rev. 2020, 120, 5517–5581; 32364373 10.1021/acs.chemrev.0c00042 PMC7730004

[7f] S. Kim , F. Loose , P. J. Chirik , Chem. Rev. 2020, 120, 5637–5681; 32458682 10.1021/acs.chemrev.9b00705

[7g] F. Masero , M. A. Perrin , S. Dey , V. Mougel , Chem. Eur. J. 2021, 27, 3892–3928; 32914919 10.1002/chem.202003134 PMC7986120

[7h] S. J. K. Forrest , B. Schluschaß , E. Y. Yuzik-Klimova , S. Schneider , Chem. Rev. 2021, 121, 6522–6587. 33973774 10.1021/acs.chemrev.0c00958

[8] For landmark articles, see:

[8a] D. V. Yandulov , R. R. Schrock , Science 2003, 301, 76–78; 12843387 10.1126/science.1085326

[8b] K. Arashiba , Y. Miyake , Y. Nishibayashi , Nat. Chem. 2011, 3, 120–125; 21258384 10.1038/nchem.906

[8c] J. S. Anderson , J. Rittle , J. C. Peters , Nature 2013, 501, 84–87; 24005414 10.1038/nature12435 PMC3882122

[8d] Y. Ashida , K. Arashiba , K. Nakajima , Y. Nishibayashi , Nature 2019, 568, 536–540. 31019315 10.1038/s41586-019-1134-2

[9] For recent reviews, see:

[9a] M. J. Chalkley , M. W. Drover , J. C. Peters , Chem. Rev. 2020, 120, 5582–5636; 32352271 10.1021/acs.chemrev.9b00638 PMC7493999

[9b] Y. Ashida , Y. Nishibayashi , Chem. Commun. 2021, 57, 1176–1189. 10.1039/d0cc07146c 33443504

[10] L. R. Doyle , A. J. Wooles , L. C. Jenkins , F. Tuna , E. J. L. McInnes , S. T. Liddle , Angew. Chem. Int. Ed. 2018, 57, 6314–6318; 10.1002/anie.201802576 PMC6003280 29633444

[11] P. Ghana , F. D. van Krüchten , T. P. Spaniol , J. van Leusen , P. Kögerler , J. Okuda , Chem. Commun. 2019, 55, 3231–3234. 10.1039/c8cc09742a 30806394

[12] For early studies of nitrogen fixation by titanium compounds, see:

[12a] M. E. Vol′pin , B. V. Shur , Nature 1966, 209, 1236;

[12b] M. Mori , J. Organomet. Chem. 2004, 689, 4210–4227;

[12c] P. J. Chirik , Organometallics 2010, 29, 1500–1517;

[12d] H. Seino , Y. Kajita , in Transition Metal-Dinitrogen Complexes, (Ed.: Y. Nishibayashi ), Wiley-VCH, Weinheim, 2019, Ch. 2, and references therein.

[13a] T. E. Hanna , E. Lobkovsky , P. J. Chirik , J. Am. Chem. Soc. 2004, 126, 14688–14689; 15535671 10.1021/ja045884r

[13b] T. E. Hanna , E. Lobkovsky , P. J. Chirik , J. Am. Chem. Soc. 2006, 128, 6018–6019; 16669654 10.1021/ja061213c

[13c] T. E. Hanna , W. H. Bernskoetter , M. W. Bouwkamp , E. Lobkovsky , P. J. Chirik , Organometallics 2007, 26, 2431–2438;

[13d] T. E. Hanna , W. H. Bernskoetter , M. W. Bouwkamp , E. Lobkovsky , P. J. Chirik , Organometallics 2009, 28, 4079–4088.

[14] For selected examples, see:

[14a] R. D. Sanner , D. M. Duggan , T. C. Mckenzie , R. E. Marsh , J. E. Bercaw , J. Am. Chem. Soc. 1976, 98, 8358–8365;

[14b] J. D. Zeinstra , J. H. Teuben , F. Jellinek , J. Organomet. Chem. 1979, 170, 39–50;

[14c] J. M. de Wolf , R. Blaauw , A. Meetsma , J. H. Teuben , R. Gyepes , V. Varga , K. Mach , N. Veldman , A. L. Spek , Organometallics 1996, 15, 4977–4983;

[14d] P. P. Fontaine , B. L. Yonke , P. Y. Zavalij , L. R. Sita , J. Am. Chem. Soc. 2010, 132, 12273–12285; 20707320 10.1021/ja100469f

[14e] T. Kurogi , Y. Ishida , H. Kawaguchi , Chem. Commun. 2013, 49, 11755–11757; 10.1039/c3cc47284a 24196319

[14f] Y. Nakanishi , Y. Ishida , H. Kawaguchi , Angew. Chem. Int. Ed. 2017, 56, 9193–9197; 10.1002/anie.201704286 28580684

[14g] A. Reinholdt , D. Pividori , A. L. Laughlin , I. D. DiMucci , S. N. MacMillan , M. G. Jafari , M. R. Gau , P. J. Carroll , J. Krzystek , A. Ozarowski , J. Telser , K. M. Lancaster , K. Meyer , D. J. Mindiola , Inorg. Chem. 2020, 59, 17834–17850; 33258366 10.1021/acs.inorgchem.0c02586 PMC7928263

[14h] D. Y. Bae , G. Lee , E. Lee , Inorg. Chem. 2021, 60, 12813–12822. 34492761 10.1021/acs.inorgchem.1c01050

[15a] R. Duchateau , S. Gambarotta , N. Beydoun , C. Bensimon , J. Am. Chem. Soc. 1991, 113, 8986–8988;

[15b] S. P. Semproni , C. Milsmann , P. J. Chirik , Organometallics 2012, 31, 3672–3682.

[16a] B. Wang , G. Luo , M. Nishiura , S. Hu , T. Shima , Y. Luo , Z. Hou , J. Am. Chem. Soc. 2017, 139, 1818–1821; 28134522 10.1021/jacs.6b13323

[16b] Z. Mo , T. Shima , Z. Hou , Angew. Chem. Int. Ed. 2020, 59, 8635–8644; 10.1002/anie.201916171 32073703

[17] G. P. Pez , P. Apgar , R. K. Crissey , J. Am. Chem. Soc. 1982, 104, 482–490.

[18] T. Shima , S. Hu , G. Luo , X. Kang , Y. Luo , Z. Hou , Science 2013, 340, 1549–1552. 23812710 10.1126/science.1238663

[19] Z.-Y. Li , Y. Li , L.-H. Mou , J.-J. Chen , Q.-Y. Liu , S.-G. He , H. Chen , J. Am. Chem. Soc. 2020, 142, 10747–10754. 32450693 10.1021/jacs.0c02021

[20a] M. Greño , E. del Horno , M. Mena , A. Pérez-Redondo , V. Varela-Izquierdo , C. Yélamos , Inorg. Chem. 2017, 56, 11220–11229; 28849636 10.1021/acs.inorgchem.7b01607

[20b] E. del Horno , J. Jover , M. Mena , A. Pérez-Redondo , C. Yélamos , Chem. Eur. J. 2019, 25, 7096–7100; 30866106 10.1002/chem.201900083

[20c] E. del Horno , J. Jover , M. Mena , A. Pérez-Redondo , C. Yélamos , Chem. Eur. J. 2022, 28, e202103085. 34735025 10.1002/chem.202103085

[21] M. García-Castro , C. García-Iriepa , E. del Horno , A. Martín , M. Mena , A. Pérez-Redondo , M. Temprado , C. Yélamos , Inorg. Chem. 2019, 58, 5314–5324. 30943022 10.1021/acs.inorgchem.9b00437

[22] E. del Horno , R. Jiménez-Aparicio , M. Mena , A. Pérez-Redondo , J. L. Priego , C. Yélamos , Inorg. Chem. 2020, 59, 3740–3752. 32101433 10.1021/acs.inorgchem.9b03399

[23a] D. F. Evans , J. Chem. Soc. 1959, 2003–2005;

[23b] S. K. Sur , J. Magn. Reson. 1989, 82, 169–173;

[23c] G. A. Bain , J. F. Berry , J. Chem. Educ. 2008, 85, 532–536.

[24] Deposition numbers 2158926 (for **3**), 2158927 (for **4**), 2158928 (for **5**) and 2158929 (for **6**) contain the supplementary crystallographic data for this paper. These data are provided free of charge by the joint Cambridge Crystallographic Data Centre and Fachinformationszentrum Karlsruhe Access Structures service.

[25] D. W. Stephan , Organometallics 1992, 11, 996–999.

[26] J. Hiller , U. Thewalt , J. Podlaha , V. Hanuš , K. Mach , Collect. Czech. Chem. Commun. 1997, 62, 1551–1561.

[27] F. Loose , M. Schmidtmann , W. Saak , R. Beckhaus , Eur. J. Inorg. Chem. 2015, 5171–5187.

[28] S. Gambarotta , C. Floriani , A. Chiesi-Villa , C. Guastini , J. Am. Chem. Soc. 1983, 105, 7295–7301.

[29] C. Lorber , Coord. Chem. Rev. 2016, 308, 76–96.

[30] For selected examples, see:

[30a] R. Duchateau , A. J. Williams , S. Gambarotta , M. Y. Chiang , Inorg. Chem. 1991, 30, 4863–4866;

[30b] K. Kaleta , P. Arndt , A. Spannenberg , U. Rosenthal , Inorg. Chim. Acta 2011, 370, 187–190;

[30c] G. B. Wijeratne , E. M. Zolnhofer , S. Fortier , L. N. Grant , P. J. Carroll , C.-H. Chen , K. Meyer , J. Krzystek , A. Ozarowski , T. A. Jackson , D. J. Mindiola , J. Telser , Inorg. Chem. 2015, 54, 10380–10397. 26451744 10.1021/acs.inorgchem.5b01796

[31] B. Hessen , J. Blenkers , J. H. Teuben , G. Helgesson , S. Jagner , Organometallics 1989, 8, 830–835.

