# Supplementary material for: Dinitrogen Binding at a Trititanium Chloride Complex and Its Conversion to Ammonia under Ambient Conditions
Source: Angew Chem Int Ed Engl. 2022 Jul 11;61(34):e202204544. doi: 10.1002/anie.202204544 (PMC9542190; doi:10.1002/anie.202204544)

checkCIF/PLATON report

You have not supplied any structure factors. As a result the full set of tests cannot be run.

THIS REPORT IS FOR GUIDANCE ONLY. IF USED AS PART OF A REVIEW PROCEDURE FOR PUBLICATION, IT SHOULD NOT REPLACE THE EXPERTISE OF AN EXPERIENCED CRYSTALLOGRAPHIC REFEREE.

No syntax errors found. CIF dictionary Interpreting this report

Datablock: 3

```
Bond precision:   C-C = 0.0055 A          Wavelength=0.71073

Cell:              a=17.249(3)     b=8.3912(15)        c=22.292(2)
                   alpha=90        beta=91.341(9)      gamma=90
Temperature:       150 K

                 Calculated                    Reported
Volume           3225.7(9)                     3225.6(9)
Space group      P 21/c                        P 21/c
Hall group       -P 2ybc                       -P 2ybc
Moiety formula   C30 H45 Cl4 Ti3               C30 H45 Cl4 Ti3
Sum formula      C30 H45 Cl4 Ti3               C30 H45 Cl4 Ti3
Mr               691.07                        691.16
Dx,g cm-3        1.423                         1.423
Z                4                             4
Mu (mm-1)        1.075                         1.075
F000             1436.0                        1436.0
F000'            1441.99
h,k,lmax         22,10,28                      22,10,28
Nref             7328                          7319
Tmin,Tmax        0.783,0.833                   0.805,0.868
Tmin'            0.764

Correction method= # Reported T Limits: Tmin=0.805 Tmax=0.868
AbsCorr = MULTI-SCAN

Data completeness= 0.999           Theta(max)= 27.395

R(reflections)= 0.0495( 5355)                          wR2(reflections)=
                                                       0.1086( 7319)
S = 1.092                   Npar= 517
```

The following ALERTS were generated. Each ALERT has the format
test-name_ALERT_alert-type_alert-level.
Click on the hyperlinks for more details of the test.

Alert level B

```
PLAT213_ALERT_2_B Atom C28            has ADP max/min Ratio .....        4.1 prolat
PLAT213_ALERT_2_B Atom C29            has ADP max/min Ratio .....        4.4 prolat
PLAT213_ALERT_2_B Atom C27'           has ADP max/min Ratio .....        4.6 prolat
PLAT213_ALERT_2_B Atom C28'           has ADP max/min Ratio .....        4.5 prolat
```

Alert level C

```
PLAT213_ALERT_2_C Atom C27            has ADP max/min Ratio .....        3.1 prolat
PLAT213_ALERT_2_C Atom C30            has ADP max/min Ratio .....        3.4 prolat
PLAT213_ALERT_2_C Atom C40            has ADP max/min Ratio .....        3.4 prolat
PLAT213_ALERT_2_C Atom C14'           has ADP max/min Ratio .....        3.8 prolat
PLAT213_ALERT_2_C Atom C16'           has ADP max/min Ratio .....        3.4 prolat
PLAT213_ALERT_2_C Atom C19'           has ADP max/min Ratio .....        3.6 prolat
PLAT213_ALERT_2_C Atom C20'           has ADP max/min Ratio .....        3.7 prolat
PLAT213_ALERT_2_C Atom C22'           has ADP max/min Ratio .....        3.1 prolat
PLAT213_ALERT_2_C Atom C23'           has ADP max/min Ratio .....        3.7 prolat
PLAT213_ALERT_2_C Atom C26'           has ADP max/min Ratio .....        3.6 prolat
PLAT213_ALERT_2_C Atom C29'           has ADP max/min Ratio .....        3.7 prolat
PLAT220_ALERT_2_C NonSolvent   Resd 1  C   Ueq(max)/Ueq(min) Range       5.3 Ratio
PLAT242_ALERT_2_C Low    'MainMol' Ueq as Compared to Neighbors of       Ti2 Check
```

Alert level G

```
PLAT003_ALERT_2_G Number of Uiso or Uij Restrained non-H Atoms ...        30 Report
PLAT005_ALERT_5_G No Embedded Refinement Details Found  in the CIF     Please Do !
PLAT083_ALERT_2_G SHELXL Second Parameter in WGHT  Unusually Large      6.11 Why ?
PLAT232_ALERT_2_G Hirshfeld Test Diff (M-X)  Ti3      --Cl4      .       6.4 s.u.
PLAT301_ALERT_3_G Main Residue  Disorder ..............(Resd  1  )       54% Note
PLAT380_ALERT_4_G Incorrectly? Oriented X(sp2)-Methyl Moiety .....       C19 Check
PLAT413_ALERT_2_G Short Inter XH3 .. XHn      H17A      ..H36B     .    2.12 Ang.
                                        1-x,-1/2+y,1/2-z  =      2_645 Check
PLAT811_ALERT_5_G No ADDSYM Analysis: Too Many Excluded Atoms ....         ! Info
PLAT860_ALERT_3_G Number of Least-Squares Restraints ............        195 Note
```

0 **ALERT level A** = Most likely a serious problem - resolve or explain
4 **ALERT level B** = A potentially serious problem, consider carefully
13 **ALERT level C** = Check. Ensure it is not caused by an omission or oversight
9 **ALERT level G** = General information/check it is not something unexpected

0 ALERT type 1 CIF construction/syntax error, inconsistent or missing data
21 ALERT type 2 Indicator that the structure model may be wrong or deficient
2 ALERT type 3 Indicator that the structure quality may be low
1 ALERT type 4 Improvement, methodology, query or suggestion
2 ALERT type 5 Informative message, check

Datablock: 4

Bond precision:	C-C = 0.0065 A		Wavelength=0.71073
Cell:	a=7.7664(7)	b=9.0858(8)	c=15.1614(12)
	alpha=94.387(6)	beta=93.311(7)	gamma=92.579(6)
Temperature:	150 K		

	Calculated	Reported
Volume	1063.62(16)	1063.62(16)
Space group	P -1	P -1
Hall group	-P 1	-P 1
Moiety formula	C28 H46 Br6 Mg O2 Ti2, C6 H14	C28 H46 Br6 Mg O2 Ti2, C6 H14
Sum formula	C34 H60 Br6 Mg O2 Ti2	C34 H60 Br6 Mg O2 Ti2
Mr	1100.27	1100.39
Dx,g cm-3	1.718	1.718
Z	1	1
Mu (mm-1)	6.053	6.053
F000	546.0	546.0
F000'	545.13	
h,k,lmax	10,11,19	10,11,19
Nref	4870	4858
Tmin,Tmax	0.258,0.357	0.443,0.549
Tmin'	0.239	

Correction method= # Reported T Limits: Tmin=0.443 Tmax=0.549
AbsCorr = MULTI-SCAN

Data completeness= 0.998 Theta(max)= 27.498

R(reflections)= 0.0393(3518) wR2(reflections)= 0.0836(4858)

S = 1.066 Npar= 240

The following ALERTS were generated. Each ALERT has the format
test-name_ALERT_alert-type_alert-level.
Click on the hyperlinks for more details of the test.

Alert level C

PLAT241_ALERT_2_C High 'MainMol' Ueq as Compared to Neighbors of C3 Check
PLAT341_ALERT_3_C Low Bond Precision on C-C Bonds .... 0.00646 Ang.

Alert level G

PLAT005_ALERT_5_G No Embedded Refinement Details Found in the CIF Please Do !
PLAT302_ALERT_4_G Anion/Solvent/Minor-Residue Disorder (Resd 2) 100% Note
PLAT302_ALERT_4_G Anion/Solvent/Minor-Residue Disorder (Resd 3) 100% Note

```
PLAT304_ALERT_4_G Non-Integer Number of Atoms in ..... (Resd  2  )      13.20 Check
PLAT304_ALERT_4_G Non-Integer Number of Atoms in ..... (Resd  3  )       6.80 Check
```

```
   0 ALERT level A = Most likely a serious problem - resolve or explain
   0 ALERT level B = A potentially serious problem, consider carefully
   2 ALERT level C = Check. Ensure it is not caused by an omission or oversight
   5 ALERT level G = General information/check it is not something unexpected

   0 ALERT type 1 CIF construction/syntax error, inconsistent or missing data
   1 ALERT type 2 Indicator that the structure model may be wrong or deficient
   1 ALERT type 3 Indicator that the structure quality may be low
   4 ALERT type 4 Improvement, methodology, query or suggestion
   1 ALERT type 5 Informative message, check
```

Datablock: 5

```
Bond precision:   C-C = 0.0055 A          Wavelength=0.71073

Cell:           a=8.8570(18)        b=10.013(3)         c=10.0873(13)
                alpha=76.397(14)    beta=68.702(14)     gamma=67.387(17)
Temperature:    150 K

                 Calculated                     Reported
Volume           764.7(3)                       764.7(3)
Space group      P -1                           P -1
Hall group       -P 1                           -P 1
Moiety formula   C32 H40 Cl2 N2 Ti2             C32 H40 Cl2 N2 Ti2
Sum formula      C32 H40 Cl2 N2 Ti2             C32 H40 Cl2 N2 Ti2
Mr               619.30                         619.36
Dx,g cm-3        1.345                          1.345
Z                1                              1
Mu (mm-1)        0.720                          0.720
F000             324.0                          324.0
F000'            324.93
h,k,lmax         11,13,13                       11,13,13
Nref             3508                           3501
Tmin,Tmax        0.902,0.931                    0.799,1.016
Tmin'            0.866

Correction method= # Reported T Limits: Tmin=0.799 Tmax=1.016
AbsCorr = MULTI-SCAN

Data completeness= 0.998           Theta(max)= 27.500
```

R(reflections)= 0.0605(2421)   wR2(reflections)= 0.1634(3501)

S = 1.086   Npar= 177

The following ALERTS were generated. Each ALERT has the format
test-name_ALERT_alert-type_alert-level.
Click on the hyperlinks for more details of the test.

Alert level G

PLAT005_ALERT_5_G No Embedded Refinement Details Found in the CIF Please Do !

---

0 **ALERT level A** = Most likely a serious problem - resolve or explain
0 **ALERT level B** = A potentially serious problem, consider carefully
0 **ALERT level C** = Check. Ensure it is not caused by an omission or oversight
1 **ALERT level G** = General information/check it is not something unexpected

0 ALERT type 1 CIF construction/syntax error, inconsistent or missing data
0 ALERT type 2 Indicator that the structure model may be wrong or deficient
0 ALERT type 3 Indicator that the structure quality may be low
0 ALERT type 4 Improvement, methodology, query or suggestion
1 ALERT type 5 Informative message, check

Datablock: 6

Bond precision: C-C = 0.0054 A   Wavelength=0.71073

Cell:	a=18.681(2)	b=18.681(2)	c=15.431(2)
	alpha=90	beta=90	gamma=120
Temperature:	150 K		

	Calculated	Reported
Volume	4663.6(12)	4663.7(12)
Space group	R -3	R -3
Hall group	-R 3	-R 3
Moiety formula	C30 H45 Cl3 N2 Ti3	C30 H45 Cl3 N2 Ti3
Sum formula	C30 H45 Cl3 N2 Ti3	C30 H45 Cl3 N2 Ti3
Mr	683.64	683.73
Dx,g cm-3	1.461	1.461
Z	6	6
Mu (mm-1)	1.033	1.033
F000	2136.0	2136.0
F000'	2144.10	
h,k,lmax	24,24,20	24,24,20
Nref	2401	2397
Tmin,Tmax	0.865,0.883	0.827,0.980
Tmin'	0.865	

Correction method= # Reported T Limits: Tmin=0.827 Tmax=0.980
AbsCorr = MULTI-SCAN

Data completeness= 0.998 Theta(max)= 27.500

R(reflections)= 0.0554(1682) wR2(reflections)= 0.1338(2397)

S = 1.067 Npar= 126

The following ALERTS were generated. Each ALERT has the format
test-name_ALERT_alert-type_alert-level.
Click on the hyperlinks for more details of the test.

Alert level G

```
PLAT005_ALERT_5_G No Embedded Refinement Details Found  in the CIF     Please Do !
PLAT083_ALERT_2_G SHELXL Second Parameter in WGHT  Unusually Large      29.42 Why ?
PLAT232_ALERT_2_G Hirshfeld Test Diff (M-X)  Ti1      --Cl1      .        5.2 s.u.
PLAT232_ALERT_2_G Hirshfeld Test Diff (M-X)  Ti1      --N1       .        8.5 s.u.
PLAT232_ALERT_2_G Hirshfeld Test Diff (M-X)  Ti1      --Cl1_a    .        5.2 s.u.
PLAT300_ALERT_4_G Atom Site Occupancy of N2         Constrained at     0.3333 Check
PLAT301_ALERT_3_G Main Residue  Disorder .............(Resd  1  )         3% Note
PLAT764_ALERT_4_G Overcomplete CIF Bond List Detected (Rep/Expd) .       1.15 Ratio
```

---

0 **ALERT level A** = Most likely a serious problem - resolve or explain
0 **ALERT level B** = A potentially serious problem, consider carefully
0 **ALERT level C** = Check. Ensure it is not caused by an omission or oversight
8 **ALERT level G** = General information/check it is not something unexpected

0 ALERT type 1 CIF construction/syntax error, inconsistent or missing data

```
 4 ALERT type 2 Indicator that the structure model may be wrong or deficient
 1 ALERT type 3 Indicator that the structure quality may be low
 2 ALERT type 4 Improvement, methodology, query or suggestion
 1 ALERT type 5 Informative message, check
```

It is advisable to attempt to resolve as many as possible of the alerts in all categories. Often the minor alerts point to easily fixed oversights, errors and omissions in your CIF or refinement strategy, so attention to these fine details can be worthwhile. In order to resolve some of the more serious problems it may be necessary to carry out additional measurements or structure refinements. However, the purpose of your study may justify the reported deviations and the more serious of these should normally be commented upon in the discussion or experimental section of a paper or in the "special_details" fields of the CIF. checkCIF was carefully designed to identify outliers and unusual parameters, but every test has its limitations and alerts that are not important in a particular case may appear. Conversely, the absence of alerts does not guarantee there are no aspects of the results needing attention. It is up to the individual to critically assess their own results and, if necessary, seek expert advice.

Publication of your CIF in IUCr journals

A basic structural check has been run on your CIF. These basic checks will be run on all CIFs submitted for publication in IUCr journals (*Acta Crystallographica*, *Journal of Applied Crystallography*, *Journal of Synchrotron Radiation*); however, if you intend to submit to *Acta Crystallographica Section C* or *E* or *IUCrData*, you should make sure that full publication checks are run on the final version of your CIF prior to submission.

Publication of your CIF in other journals

Please refer to the *Notes for Authors* of the relevant journal for any special instructions relating to CIF submission.

PLATON version of 13/07/2021; check.def file version of 13/07/2021

Datablock 3 - ellipsoid plot

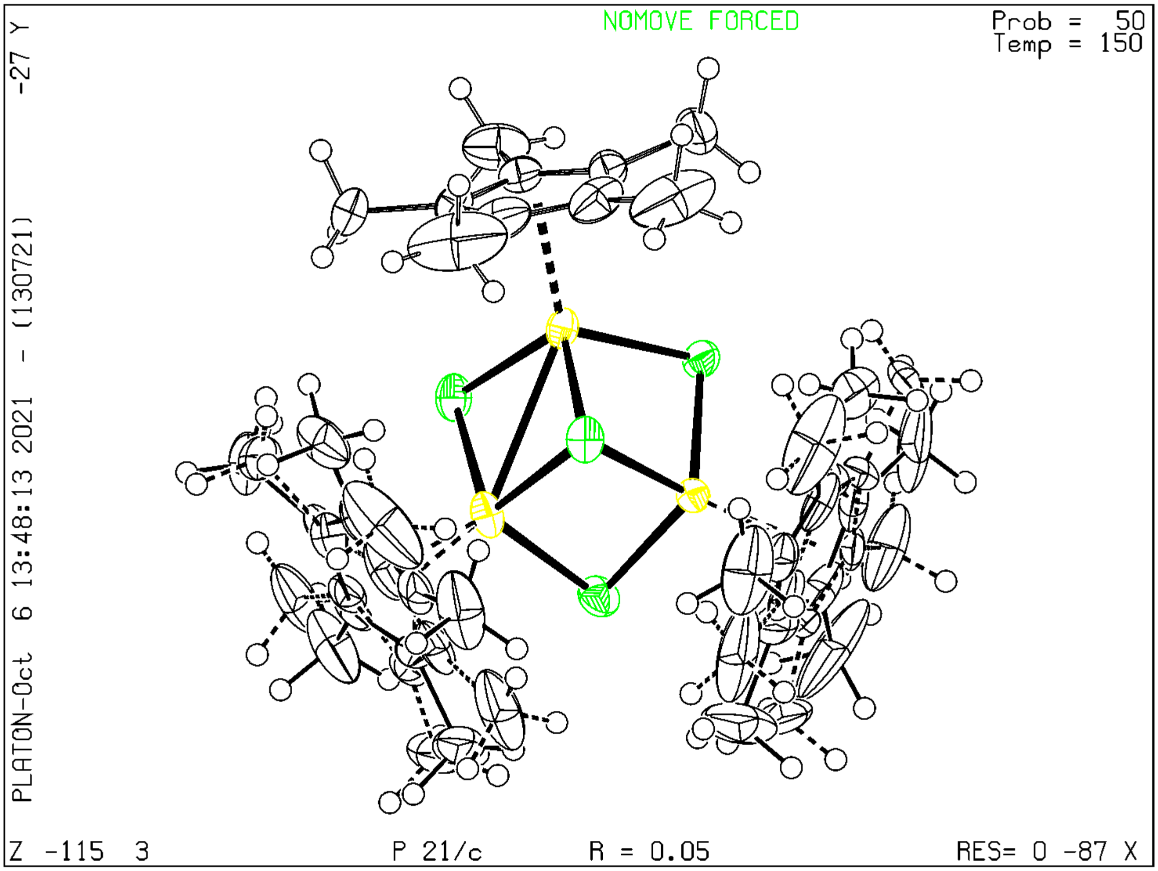

Datablock 4 - ellipsoid plot

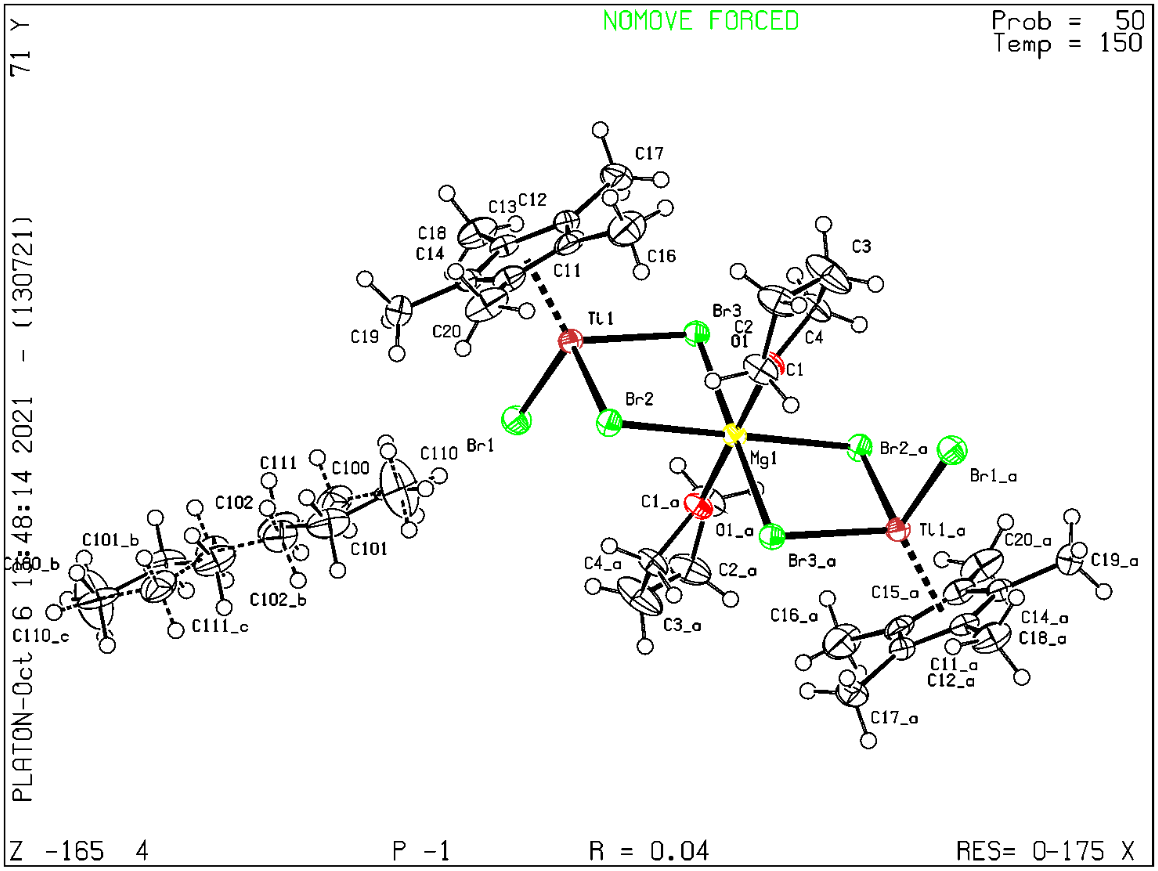

Datablock 5 - ellipsoid plot

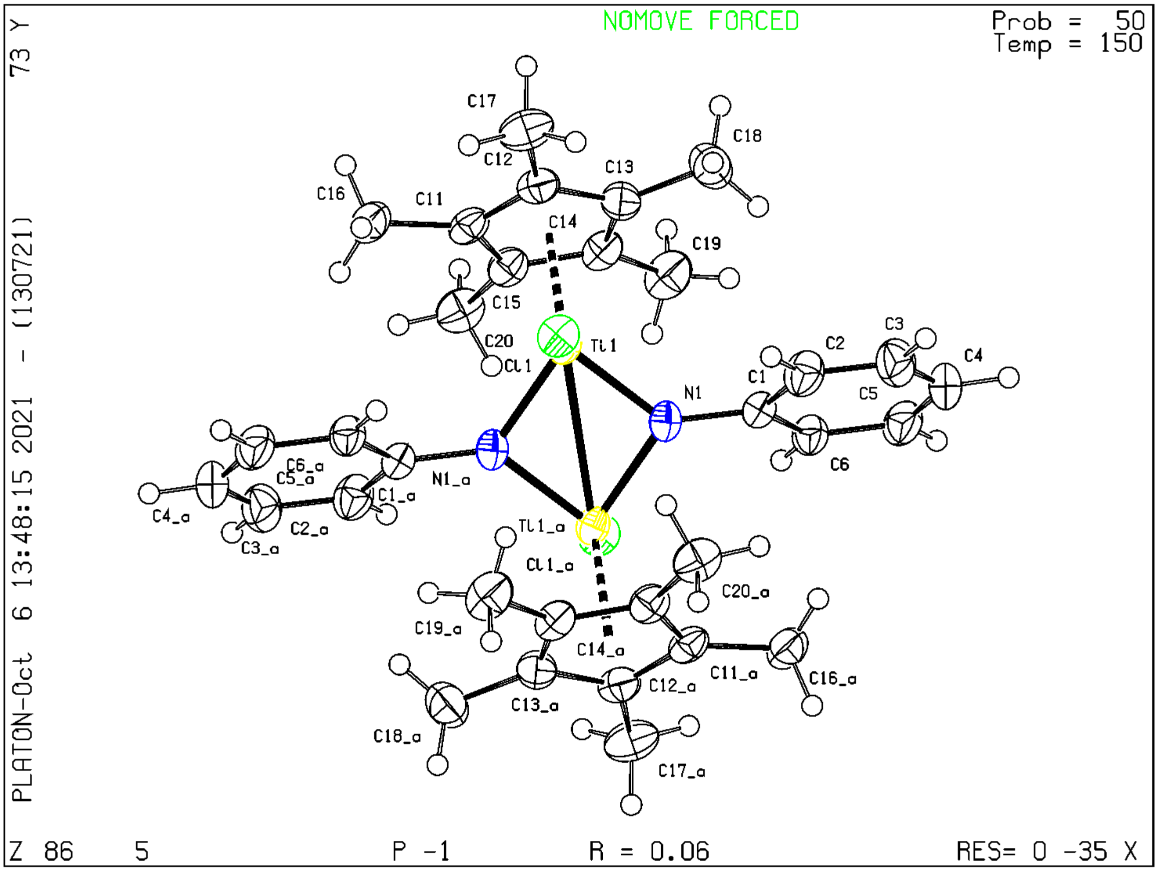

Datablock 6 - ellipsoid plot

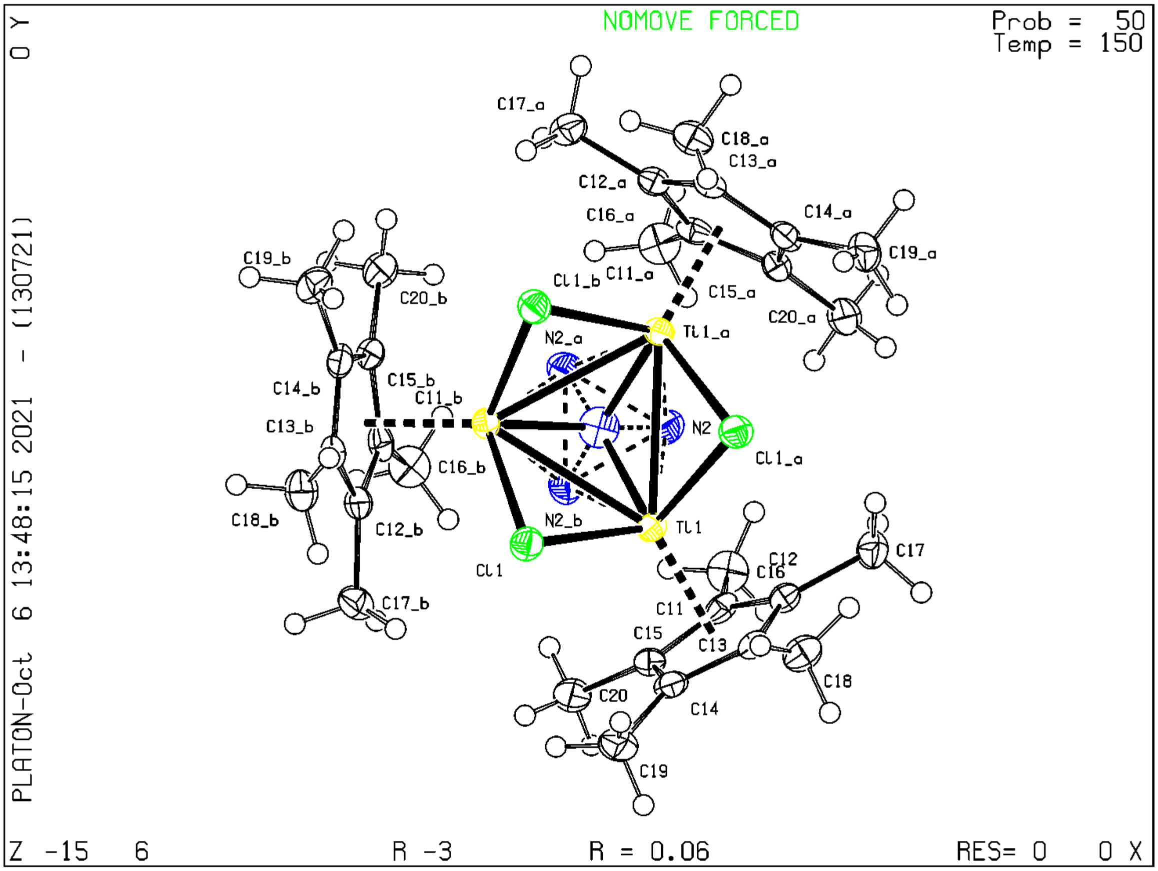

Supplement: Supplementary file 2 — Supporting Information [file ANIE-61-0-s002.pdf]
