# Supplementary material for: Dinitrogen Binding at a Trititanium Chloride Complex and Its Conversion to Ammonia under Ambient Conditions
Source: Angew Chem Int Ed Engl. 2022 Jul 11;61(34):e202204544. doi: 10.1002/anie.202204544 (PMC9542190; doi:10.1002/anie.202204544)
Supplement: Supplementary file 3 — Supporting Information [file ANIE-61-0-s003.pdf]

Angewandte Chemie

Eine Zeitschrift der Gesellschaft Deutscher Chemiker

Supporting Information

Dinitrogen Binding at a Trititanium Chloride Complex and Its Conversion to Ammonia under Ambient Conditions

E. del Horno, J. Jover, M. Mena, A. Pérez-Redondo, C. Yélamos**

Supporting Information

Contents:

- Experimental details and characterization data.
- Schemes S1 and S2.
- Experimental crystallographic data of compounds **3**–**6**.
- Perspective views of the crystal structure of compounds **4** and **5**.
- Tables for selected lengths and angles for compounds **3**–**6**.
- Selected figures of computed orbitals of compounds **3**, **6** and **6´**.
- Photograph of the experimental setup for the cyclic preparation of NH_4Cl.
- Selected ^{1}H, $^{13}C\{^{1}H\}$, and ^{15}N NMR spectra.
- IR spectra of **6** and **6-^{15}N**.
- Cartesian coordinates of compounds **3**, **6** and **6´**.

Experimental Details and Characterization Data:

General Considerations. All manipulations were carried out under argon atmosphere using Schlenk line or glovebox techniques. Toluene and hexane were distilled from Na/K alloy just before use. Tetrahydrofuran was distilled from purple solutions of sodium benzophenone just prior to use. NMR solvents were dried with Na/K alloy ([D_6]benzene, [D_8]tetrahydrofuran) or CaH_2 ([D_1]chloroform, [D_6]DMSO) and distilled before use. Oven-dried glassware was repeatedly evacuated with a pumping system (ca. 1×10^{-3} Torr) and subsequently filled with inert gas. Azobenzene was purchased from Aldrich and used as received. Magnesium (99.5%) was purchased from Scharlau and used as received. Nitrogen (≥ 99.9999%, H_2O < 0.5 ppm, O_2 < 0.5 ppm) and hydrogen chloride (≥ 99.999%, H_2O < 0.5 ppm, O_2 < 0.5 ppm) were purchased from Linde and used as received. 15-Nitrogen (+98%) was purchased from Cambridge Isotope Laboratories and used as received. [Ti(η^5-C_5Me_5)X_3] (X = Cl, Br) were prepared according to published procedures.[1]

Samples for infrared spectroscopy were prepared as KBr pellets, and the spectra were obtained using a Perkin-Elmer FT-IR-Frontier or Bruker FT-IR-ALPHA II spectrophotometers. ^1H, $^{13}C\{^1H\}$, and ^{15}N NMR spectra were recorded on a Varian Mercury-300 or Unity-500 spectrometers. Chemical shifts (δ, ppm) in the ^1H, $^{13}C\{^1H\}$ NMR spectra are given relative to residual protons or to carbon of the solvent, [D_6]benzene (^1H: δ = 7.15; ^{13}C: δ = 128.0), [D_8]tetrahydrofuran (^1H: δ = 3.58; ^{13}C: δ = 67.2), [D_1]chloroform (^1H: δ = 7.24; ^{13}C: δ = 77.0) or [D_6]DMSO (^1H: δ = 2.49; ^{13}C: δ = 39.0). Chemical shifts (δ, ppm) in the ^{15}N NMR spectra are given relative to $MeNO_2$ as external reference. The effective magnetic moments were determined by the Evans NMR method at 293 K (using a 300 MHz instrument with a field strength of 7.05 Tesla).[2] Electron impact mass spectra were obtained

at 70 eV on a Thermo Scientific ITQ 900 mass spectrometer. Microanalyses (C, H, N) were performed in a Perkin Elmer CHNS/O 2400 or Leco CHNS-932 microanalyzers.

Synthesis of [{Ti(η^5-C_5Me_5)Cl}$_3$(μ_3-Cl)] (3). A 150 mL Schlenk tube was charged with [Ti(η^5-C_5Me_5)Cl_3] (1.00 g, 3.45 mmol), magnesium (0.084 g, 3.45 mmol), and tetrahydrofuran (50 mL). The reaction mixture was stirred at room temperature for 20 h. The volatile components of the resultant solution were removed under reduced pressure to give a brown solid. This solid was extracted with toluene (30 mL) and, after filtration, the volatile components of the filtrate were removed under reduced pressure to give **3** as a brown solid (0.69 g, 87%). IR (KBr, cm^{-1}): $\tilde{\nu}$ 2969 (m), 2905 (vs), 2856 (s), 1487 (w), 1430 (m), 1375 (vs), 1261 (w), 1023 (m), 792 (m), 727 (w), 620 (w), 450 (w), 397 (vs). ^{1}H NMR (300 MHz, [D_6]benzene, 20 ºC, δ): 10.97 (s br., $\Delta\nu_{1/2}$ = 50 Hz, C_5Me_5). ^{1}H NMR (300 MHz, [D_8]tetrahydrofuran, 20 ºC, δ): 10.94 (s br., $\Delta\nu_{1/2}$ = 49 Hz, C_5Me_5). MS (EI, 70 eV): m/z (%) 691 (24) [M]$^+$, 508 (9) [Ti(C_5Me_5)Cl_2]$_2^+$, 471 (35) [M-Ti(C_5Me_5)Cl]$^+$, 436 (58) [M-Ti(C_5Me_5)Cl_2]$^+$, 253 (26) [Ti(C_5Me_5)Cl_2]$^+$, 218 (100) [Ti(C_5Me_5)Cl]$^+$. Anal. Calcd for $C_{30}H_{45}Cl_4Ti_3$ (*M*w = 691.09): C 52.14, H 6.56. Found: C 51.98, H 6.52. The effective magnetic moment of **3** was determined to be 2.17 μ_B (based on a unit formula of $C_{30}H_{45}Cl_4Ti_3$) on a [D_6]benzene solution.

Synthesis of [{Ti(η^5-C_5Me_5)Br(μ-Br)$_2$}$_2$Mg(thf)$_2$] (4). *Method A:* A 150 mL Schlenk tube was charged with [Ti(η^5-C_5Me_5)Br_3] (0.30 g, 0.71 mmol), magnesium (0.019 g, 0.78 mmol), and tetrahydrofuran (30 mL). The reaction mixture was stirred at room temperature for 20 h to give a brown solution. The volatile compounds of the solution were removed under reduced pressure. The resultant solid was extracted with toluene (30 mL), filtered and the volatile components of the filtrate were removed under reduced pressure to give **4** as a brown-

green solid (0.14 g, 39%). *Method B:* A 150 mL Schlenk tube was charged with [{Ti(η^5-C_5Me_5)Br_3] (0.60 g, 1.42 mmol), magnesium (0.017 g, 0.70 mmol), and tetrahydrofuran (30 mL). The reaction mixture was stirred at room temperature for 20 h to give a brown solution. The volatile compounds of the solution were removed under reduced pressure. The resultant solid was extracted with toluene (30 mL), filtered and the volatile components of the filtrate were removed under reduced pressure to give **4** as a brown-green solid (0.55 g, 77%). Crystallization in *n*-hexane at –35 ºC gave green crystals of **4**·C_6H_{14} (0.045 g, 6%), which were used for X-ray diffraction and elemental analysis. IR (KBr, cm^{-1}): $\tilde{\nu}$ 2978 (m), 2963 (m), 2903 (s), 1487 (w), 1457 (m), 1378 (m), 1261 (m), 1097 (w), 1024 (vs), 873 (m), 802 (m), 675 (w), 661 (w), 549 (w), 445 (w), 427 (w). Anal. Calcd for $C_{34}H_{60}Br_6MgO_2Ti_2$ (*M*w = 1100.30): C 37.11, H 5.50. Found: C 37.23, H 5.51. The effective magnetic moment of **4** was determined to be 2.25 μ_B (based on a unit formula of $C_{34}H_{60}Br_6MgO_2Ti_2$) on a [$D_6$]benzene solution.

Synthesis of [{Ti(η^5-C_5Me_5)Cl(μ-NPh)}$_2$] (5). *Method A:* A 150 mL Schlenk tube was charged with **3** (0.30 g, 0.43 mmol), azobenzene (0.12 g, 0.66 mmol), and toluene (20 mL). The reaction mixture was stirred at room temperature for 20 h. After filtration, the volatile components of the solution were removed under reduced pressure to give a brown-orange solid. This solid was washed with hexane (10 mL) and was vacuum-dried to afford **5** as a brown solid (0.16 g, 48%). *Method B:* A 150 mL Schlenk tube was charged with **6** (0.30 g, 0.44 mmol), azobenzene (0.12 g, 0.66 mmol), and hexane (10 mL). The reaction mixture was stirred at room temperature for 20 h to give a brown suspension. The solid was isolated by filtration onto a glass frit and vacuum-dried to afford **5** as a brown solid (0.19 g, 47%). IR (KBr, cm^{-1}): $\tilde{\nu}$ 3070 (w), 3048 (w), 3023 (w), 3005 (w), 2980 (w), 2955 (w), 2906 (m), 2856

(m), 1582 (s), 1569 (w), 1476 (vs), 1427 (m), 1385 (w), 1240 (vs), 1068 (w), 1029 (w), 888 (w), 761 (vs), 698 (s), 623 (s), 576 (vs), 516 (s), 463 (s). ^{1}H NMR (300 MHz, [D$_6$]benzene, 20 ºC, δ): 7.04–6.73 (m, 10H; C_6H_5), 1.75 (s, 30H; C_5Me_5) ^{13}C{^{1}H} NMR (75 MHz, [D$_6$]benzene, 20 ºC, δ): 155.7, 128.5, 127.4, 124.1 (C_6H_5), 123.1 (C_5Me$_5$), 11.6 (C$_5$*Me*$_5$). Anal. Calcd for $C_{32}H_{40}Cl_2N_2Ti_2$ (*M*w = 619.31): C 62.06, H 6.51, N 4.52. Found: C 61.74, H 6.61, N 4.19.

Synthesis of [{Ti(η^5-C_5Me_5)(μ-Cl)}$_3$(μ_3-η^1:η^2:η^2-N_2)] (6). A 150 mL ampule (Teflon stopcock) was charged with [Ti(η^5-C_5Me_5)Cl_3] (1.00 g, 3.45 mmol), magnesium (0.084 g, 3.46 mmol), and tetrahydrofuran (30 mL). The reaction mixture was stirred at room temperature for 16 h. After it was cooled to –78 ºC, the argon atmosphere was changed by dinitrogen and the brown solution was stirred at room temperature for 16 h. Then the volatile components of the resultant solution were removed under reduced pressure to give a brown solid. This solid was extracted with toluene (30 mL), and after filtration and elimination of the volatile components of the filtrate under reduced pressure, complex **6** was isolated as a brown solid (0.69, 87%). The preparation of the ^{15}N-enriched compound [{Ti(η^5-C_5Me_5)Cl}$_3$(μ_3-η^1:η^2:η^2-$^{15}N_2$)] (**6-^{15}N**) was carried out in exactly the same manner (72% yield) as for the parent complex **6**. Samples of compounds **6** and **6-^{15}N** were stored under an argon atmosphere at low temperatures (–35 ºC).

Complex **6**: IR (KBr, cm^{-1}): $\tilde{\nu}$ 2970 (m), 2948 (w), 2907 (vs), 2854 (s), 2721 (w), 1493 (m), 1430 (s), 1374 (vs), 1277 (s) (ν_{NN}), 1162 (w), 1067 (w), 1025 (s), 790 (vs), 772 (s), 729 (m), 694 (w), 653 (w), 622 (w), 599 (w), 549 (w), 459 (m), 408 (m). ^{1}H NMR (300 MHz, [D$_6$]benzene, 20 ºC, δ): 1.87 (s, 30H; C_5Me_5), 1.70 (s, 15H; C_5Me_5). ^{13}C{^{1}H} NMR (75 MHz, [D$_6$]benzene, 20 ºC, δ): 121.2 (C_5Me$_5$), 120.6 (C_5Me$_5$), 12.4 (C$_5$*Me*$_5$), 12.2 (C$_5$*Me*$_5$). ^{1}H NMR

(300 MHz, $[D_8]$tetrahydrofuran, 20 ºC, δ): 1.81 (s, 30H; C_5Me_5), 1.76 (s, 15H; C_5Me_5). $^{13}C\{^1H\}$ NMR (75 MHz, $[D_8]$tetrahydrofuran, 20 ºC, δ): 121.4 (C_5Me_5), 121.0 (C_5Me_5), 12.1 ($C_5\mathit{Me}_5$), 12.0 ($C_5\mathit{Me}_5$). Anal. Calcd for $C_{30}H_{45}Cl_3N_2Ti_3$ (*M*w = 683.65): C 52.71, H 6.63, N 4.10. Found: C 52.51, H 6.77, N 4.01.

Complex **6-^{15}N**: IR (KBr, cm^{-1}): $\tilde{\nu}$ 2972 (m), 2904 (vs), 2855 (s), 2721 (w), 1494 (m), 1429 (s), 1375 (vs), 1261 (w), 1234 (w) (ν_{NN}), 1164 (w), 1108 (w), 1068 (w), 1025 (vs), 921 (w), 879 (m), 790 (vs), 771 (s), 729 (m), 694 (w), 621(w), 465 (w), 451 (w), 436 (w). ^1H NMR (500 MHz, $[D_6]$benzene, 20 ºC, δ): 1.87 (s, 30H; C_5Me_5), 1.70 (s, 15H; C_5Me_5). ^{15}N NMR (50.7 MHz, $[D_6]$benzene, 20 ºC, δ): 95.4 (d, $^1J(^{15}N,^{15}N)$ = 19.8 Hz; μ_3-η^1:η^2:η^2-*N*N), –0.4 (d, $^1J(^{15}N,^{15}N)$ = 19.8 Hz; μ_3-η^1:η^2:η^2-N*N*).

Reaction of 6-$^{15}N_2$ with HCl. A 5 mm valved NMR tube (Teflon stopcock) was charged with a solution of **6-$^{15}N_2$** (0.010 g, 0.015 mmol) in $[D_6]$benzene (1.00 mL). After cooling at –78 ºC, the argon atmosphere was changed by hydrogen chloride to immediately give an orange suspension. The volatile components were removed under reduced pressure and the resultant orange solid was dissolved in $[D_6]$DMSO. The ^1H NMR spectrum revealed the expected resonance signal for $^{15}NH_4Cl$: ^1H NMR (300 MHz, $[D_6]$DMSO, 20 ºC, δ): 7.45 (d, $^1J(^1H,^{15}N)$ = 71 Hz; $^{15}NH_4Cl$).

Reaction of 6 with HCl. A 150 mL ampule (Teflon stopcock) was charged with $[Ti(\eta^5$-$C_5Me_5)Cl_3]$ (1.00 g, 3.45 mmol), magnesium (0.084 g, 3.46 mmol), and tetrahydrofuran (30 mL). The reaction mixture was stirred at room temperature for 16 h. After it was cooled to –78 ºC, the argon atmosphere was changed by dinitrogen, and the brown solution was stirred at room temperature for 16 h. Then it was cooled to –78 ºC and the dinitrogen atmosphere was changed by hydrogen chloride. Immediately, the reaction gave an orange suspension that

was stirred at room temperature for 16 hours. After centrifugation, the solid was isolated by filtration, washed with tetrahydrofuran (3 × 5 mL) and vacuum-dried to give NH_4Cl as a white powder (0.106 g, 86%). On the other hand, the volatile components of the tetrahydrofuran filtrate were removed under reduced pressure, and the addition of toluene (30 mL) led to an orange solution and a white solid of [$MgCl_2(thf)_2$]. After a new filtration, the solvent of the filtrate were removed to afford [Ti(η^5-C_5Me_5)Cl_3] as a red powder (0.75 g, 75%).

Spectroscopic data for [Ti(η^5-C_5Me_5)Cl_3]: ^{1}H NMR (300 MHz, [D_1]chloroform, 20 ºC, δ): 2.36 (s, C_5Me_5).

Spectroscopic data for NH_4Cl: ^{1}H NMR (300 MHz, [D_6]DMSO, 20 ºC, δ): 7.39 (t, $^1J(^1H,^{14}N)$ = 50 Hz; NH_4Cl).

Cyclic preparation of NH_4Cl. The photograph of the experimental setup is shown in Figure S7. In an argon-filled glovebox, a 150 mL glass ampule (Teflon stopcock) was charged with [Ti(η^5-C_5Me_5)Cl_3] (0.25 g, 0.86 mmol), magnesium (0.63 g, 25.9 mmol), tetrahydrofuran (60 mL), and a magnetic stir bar. The ampule was taken out of the glovebox and was joined by flexible tubing to a three-way glass stopcock connected by one position to a dinitrogen-vacuum Schlenk line and by the other way to a gas cylinder of HCl equipped with a pressure regulator. After cooling at –78 ºC, the argon atmosphere was removed under vacuum and changed by a dinitrogen atmosphere (ca. 1 atm). The reaction mixture was stirred at room temperature for 1 h. After cooling again at –78 ºC, the dinitrogen atmosphere was removed under vacuum and changed by a HCl atmosphere (ca. 1 atm), and the reaction mixture was stirred at room temperature for 15 min to give an orange suspension. This cycle was repeated for additional eight times (N_2 atmosphere for 30 min / HCl atmosphere for 15 min). After

that, the reaction mixture was stirred under a HCl atmosphere for 16 h to eliminate the magnesium excess as [$MgCl_2(thf)_2$] and the resultant orange suspension was cooled to 4 ºC for 3 days. The solid was isolated by filtration and vacuum-dried to give NH_4Cl as a white powder (0.20 g, 3.75 mmol, 72% based on nine completed reactions of the titanium complex since each completed cycle should produce 0.57 mmol, 0.031 g of NH_4Cl).

X-ray structure determination of 3, 4, 5 and 6. Brown crystals of [{Ti(η^5-C_5Me_5)Cl}$_3$(μ_3-Cl)] (**3**) were grown from a toluene solution at –35 ºC. Green crystals of [{Ti(η^5-C_5Me_5)Br(μ-Br)$_2$}$_2$Mg(thf)$_2$]·C_6H_{14} (**4**·C_6H_{14}) were grown from a *n*-hexane solution at –35 ºC. Brown crystals of [{Ti(η^5-C_5Me_5)Cl(μ-NPh)}$_2$] (**5**) were grown from a hexane solution at room temperature. Brown crystals of [{Ti(η^5-C_5Me_5)(μ-Cl)}$_3$(μ_3-η^1:η^2:η^2-N_2)] (**6**) were grown from a toluene/hexane solution at –35 ºC. The crystals were removed from the Schlenk tubes and covered with a layer of a viscous perfluoropolyether (Fomblin®Y). A suitable crystal was selected with the aid of a microscope, located on a micromount, and immediately placed in the low temperature nitrogen stream of the diffractometer. The intensity data sets were collected at 150 K on a Bruker-Nonius KappaCCD diffractometer equipped with an Oxford Cryostream 700 unit.

Crystallographic data for complexes **3**-**6** are presented in Table S1. The structures were solved, using the WINGX package,[3] by direct (**6**) (SHELXS-2013)[4] or intrinsic phasing methods (**3**-**5**) (SHELXT),[5] and refined by least-squares against F^2 (SHELXL-2014/7).[4] Crystals of **3** showed disorder for the pentamethylcyclopentadienyl rings linked to Ti(1) and Ti(2). These disorders were conventionally treated by using the PART tool of the SHELXL program and allowing free refinement of the occupancy factor with the FVAR command. The final values of occupancy were 53.8 and 46.2% for C(11)–C(20), and 65 and 35% for

C(21)–C(30). All non-hydrogen atoms were anisotropically refined, while all the hydrogen atoms were positioned geometrically and refined by using a riding model. Additionally, the carbon atoms of the C_5Me_5 ligand linked to Ti(2) (C(21)–C(30) and C(21)'–C(30)') were restrained with DELU and SIMU instructions. Furthermore, DELU restraints were also employed for the carbon atoms C(11)'–C(20)' linked to Ti(1).

Complex **4** crystallized with a molecule of hexane which presented disorder. This disorder was also treated by using the PART tool with final values of 66 and 34% for carbon atoms C(100)-C(102) and C(110)-C(112) respectively. In the crystallographic study of **4** and **5**, all non-hydrogen atoms were anisotropically refined, while the hydrogen atoms were positioned geometrically and refined by using a riding model.

Finally, complex **6** crystallized in *R*–3 space group. The asymmetric unit was made up by a third of the molecule with a tertiary axis crossing through the nitrogen atom N(1). However, the second nitrogen atom N(2) was located out of this axis, so three different positions for N(2) were generated by using this symmetry element. Then, the occupancy for N(2) was constrained to be 33.33%. All non-hydrogen atoms were anisotropically refined, whereas the hydrogen atoms were placed geometrically and refined by using a riding model.

Computational Details. All the structures have been fully optimized in gas phase employing the Gaussian09 suite of programs,[6] and using the unrestricted formalism of the B3LYP functional.[7] All the C, Cl, N, H and Ti atoms are described with the triple-ζ all electron basis set of Ahlrichs and co-workers.[8] The computed energies have been corrected with the D3 empirical dispersion method of Grimme.[9] All the open-shell computed structures show a certain degree of spin contamination by states with higher spin multiplicity. However, complexes **3** and **6**, as reported in the main text, show only minor spin contamination

contributions. For these compounds S^2 values of 0.7548 and 0.1830 are obtained for compounds **3** and **6**, respectively. These values differ only slightly from the expected values for non-contaminated species, which should be of 0.75 for **3** and 0.00 for **6**.

The NBO analysis has been carried out at the same level of theory as above with the NBO 3.1 program included in Gaussian09.[10] This procedure has been carried out with the standard NBO keywords, with the only exception of printing the NBO bond indexes via the "$NBO PLOT BNDIDX $END" command.

References

(1) a) G. Hidalgo Llinás, M. Mena, F. Palacios, P. Royo, R. Serrano, *J. Organomet. Chem.* **1988**, *340*, 37–40; b) G. Hidalgo Llinás, M. Mena, F. Palacios, P. Royo, R. Serrano, *Synthethic Methods of Organometallic and Inorganic Chemistry, Brauer/Herrman, vol. 1*; Thieme Verlag, Stuttgart **1996**, 95–97.

(2) a) D. F. Evans, *J. Chem. Soc.* **1959**, 2003–2005; b) S. K. Sur, *J. Magn. Reson.* **1989**, 169–173; c) G. A. Bain, J. F. Berry, *J. Chem. Educ.* **2008**, *85*, 532–536.

(3) L. J. Farrugia, *J. Appl. Crystallogr.* **2012**, *45*, 849–854.

(4) G. M. Sheldrick, *Acta Crystallogr., Sect. C* **2015**, *71*, 3–8.

(5) G. M. Sheldrick, *Acta Crystallogr., Sect. A* **2015**, *71*, 3–8.

(6) M. J. Frisch, G. W. Trucks, H. B. Schlegel, G. E. Scuseria, M. A. Robb, J. R. Cheeseman, G. Scalmani, V. Barone, B. Mennucci, G. A. Petersson, H. Nakatsuji, M. Caricato, X. Li, H. P. Hratchian, A. F. Izmaylov, J. Bloino, G. Zheng, J. L. Sonnenberg, M. Hada, M. Ehara, K. Toyota, R. Fukuda, J. Hasegawa, M. Ishida, T. Nakajima, Y. Honda, O. Kitao, H. Nakai, T.

Vreven, J. J. A. Montgomery, J. E. Peralta, F. Ogliaro, M. Bearpark, J. J. Heyd, E. Brothers, K. N. Kudin, V. N. Staroverov, R. Kobayashi, J. Normand, K. Raghavachari, A. Rendell, J. C. Burant, S. S. Iyengar, J. Tomasi, M. Cossi, N. Rega, N. J. Millam, M. Klene, J. E. Knox, J. B. Cross, V. Bakken, C. Adamo, J. Jaramillo, R. Gomperts, R. E. Stratmann, O. Yazyev, A. J. Austin, R. Cammi, C. Pomelli, J. W. Ochterski, R. L. Martin, K. Morokuma, V. G. Zakrzewski, G. A. Voth, P. Salvador, J. J. Dannenberg, S. Dapprich, A. D. Daniels, Ö. Farkas, J. B. Foresman, J. V. Ortiz, J. Cioslowski, D. J. Fox, *Gaussian09, Revision D.01*, Gaussian, Inc.: Wallingford CT, **2009**.

(7) a) A. D. Becke, *J. Chem. Phys.* **1993**, 98, 5648–5652; b) C. Lee, W. Yang, R. G. Parr, *Phys. Rev. B* **1988**, *37*, 785–789; c) B. Miehlich, A. Savin, H. Stoll, H. Preuss, *Chem. Phys. Lett.* **1989**, *157*, 200–206.

(8) A. Schäfer, C. Huber, R. Ahlrichs, *J. Chem. Phys.* **1994**, *100*, 5829–5835.

(9) S. Grimme, J. Antony, S. Ehrlich, H. Krieg, *J. Chem. Phys.* **2010**, *132*, 154104–154119.

(10) E. D. Glendening, A. E. Reed, J. E. Carpenter, F. Weinhold, *NBO*, Version 3.1.

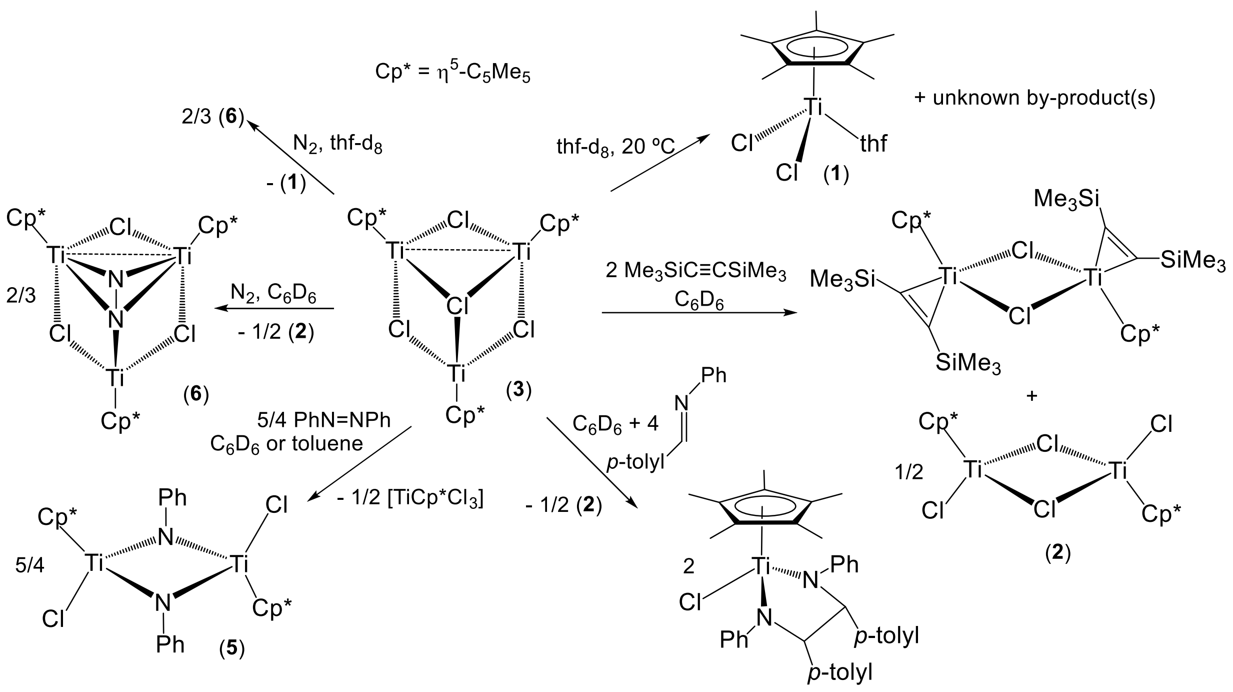

Scheme S1. Reactivity of [{TiCp*(μ-Cl)}3(μ3-Cl)] (**3**).

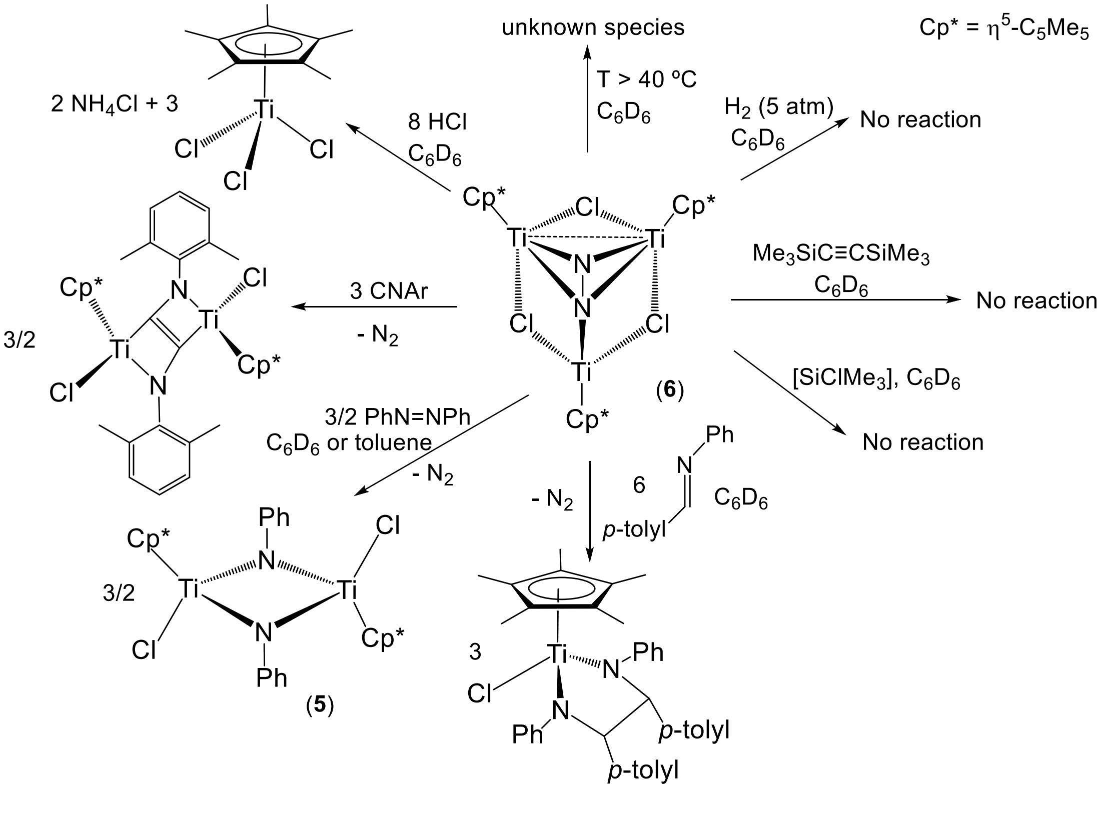

Scheme S2. Reactivity of [{TiCp*(μ-Cl)}3(μ3-η1:η2:η2-N2)] (**6**).

Table S1. Crystallographic data for complexes **3**, **4**·C_6H_{14}, **5** and **6**.

Compound	**3**	**4**·C_6H_{14}	**5**	**6**
Empirical formula	$C_{30}H_{45}Cl_4Ti_3$	$C_{34}H_{60}Br_6MgO_2Ti_2$	$C_{32}H_{40}Cl_2N_2Ti_2$	$C_{30}H_{45}Cl_3N_2Ti_3$
Formula weight	691.16	1100.39	619.36	683.73
Temperature (K)	150(2)	150(2)	150(2)	150(2)
Wavelength (Å)	0.71073	0.71073	0.71073	0.71073
Crystal system	Monoclinic	Triclinic	Triclinic	Trigonal
Space group	$P2_1/c$	*P*–1	*P*–1	*R*–3
a(Å); *α*(°)	17.249(3)	7.766(1); 94.39(1)	8.857(2); 76.40(1)	18.681(2)
b(Å); *β*(°)	8.391(2); 91.34(1)	9.086(1); 93.31(1)	10.013(3); 68.70(1)	18.681(2)
c(Å); *γ*(°)	22.292(2)	15.161(1); 92.58(1)	10.087(1); 67.39(2)	15.431(2)
Volume ($Å^3$)	3225.6(9)	1063.6(2)	764.7(3)	4663.7(12)
Z	4	1	1	6
Density (calculated) (Mg/m^3)	1.423	1.718	1.345	1.461
Absorption coefficient (mm^{-1})	1.075	6.053	0.720	1.033
F(000)	1436	546	324	2136
Crystal size (mm^3)	0.25 × 0.19 × 0.17	0.23 × 0.20 × 0.17	0.20 × 0.12 × 0.10	0.14 × 0.12 × 0.12
Theta range for data collection	3.02 to 27.40º	3.03 to 27.50º	3.28 to 27.50°	3.58 to 27.50º
Index ranges	–22 to 22,<br>–10 to 10,<br>–28 to 28	–10 to 10,<br>–11 to 11,<br>–19 to 19	–11 to 11,<br>–13 to 13,<br>–13 to 12	–24 to 24,<br>–24 to 24,<br>–18 to 20
Reflections collected	76638	37866	22514	20113
Independent reflections	7319 [R(int) = 0.072]	4858 [R(int) = 0.094]	3501 [R(int) = 0.107]	2397 [R(int) = 0.113]
Reflections [I>2σ(I)]	5355	3518	2421	1682
Completeness to theta	99.8%	99.8%	99.7%	99.8%
Goodness-of-fit on F^2	1.092	1.066	1.086	1.061
Final R^a indices [I>2σ(I)]	R1 = 0.050,<br>wR2 = 0.095	R1 = 0.039,<br>wR2 = 0.072	R1 = 0.060,<br>wR2 = 0.139	R1 = 0.055,<br>wR2 = 0.110
R indices (all data)	R1 = 0.082,<br>wR2 = 0.109	R1 = 0.074,<br>wR2 = 0.084	R1 = 0.101,<br>wR2 = 0.163	R1 = 0.097,<br>wR2 = 0.129
Largest diff. peak and hole (e $Å^{-3}$)	0.604 and –0.394	1.042 and –0.569	0.895 and –0.614	0.627 and –0.435

^{a} $R1 = \Sigma||F_0|-|F_c||/[\Sigma|F_0|]$, $wR2 = \{[\Sigma w(F_0^2-F_c^2)^2] / [\Sigma w(F_0^2)^2]\}^{1/2}$

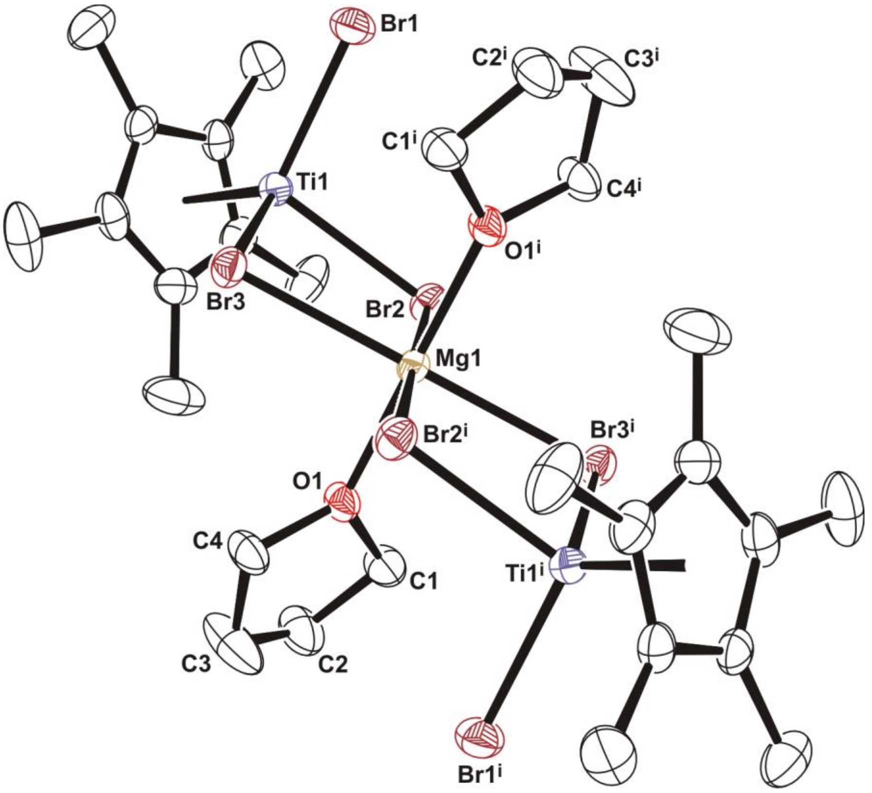

Figure S1. Perspective view of complex **4**·C_6H_{14} (thermal ellipsoids at the 50% probability level). The *n*-hexane solvent molecule and hydrogen atoms of the η^5-C_5Me_5 and thf groups are omitted for clarity. Symmetry code: (i) 1 – *x*, 2 – *y*, 1 – *z*.

Table S2. Selected Lengths (Å) and Angles (deg) for **4**·C_6H_{14}.

Ti(1)-Br(1)	2.441(1)	Ti(1)-Br(2)	2.573(1)
Ti(1)-Br(3)	2.588(1)	Mg(1)-Br(2)	2.665(1)
Mg(1)-Br(3)	2.666(1)	Mg(1)-O(1)	2.098(3)
Ti(1)-Cm(1)[a]	2.005		
Br(1)-Ti(1)-Br(2)	101.2(1)	Br(1)-Ti(1)-Br(3)	105.1(1)
Br(2)-Ti(1)-Br(3)	88.2(1)	Br(2)-Mg(1)-Br(3)	84.7(1)
Br(2)-Mg(1)-O(1)	89.6(1)	Br(3)-Mg(1)-O(1)	90.3(1)
Br(2)-Mg(1)-O(1)^i	90.4(1)	Br(3)-Mg(1)-O(1)^i	89.7(1)
Br(2)-Mg(1)-Br(3)^i	95.3(1)	Ti(1)-Br(2)-Mg(1)	92.9(1)
Ti(1)-Br(3)-Mg(1)	92.6(1)		

[a] Cm = Centroid of the η^5-C_5Me_5 ring. Symmetry code: (i) 1 – *x*, 2 – *y*, 1 – *z*.

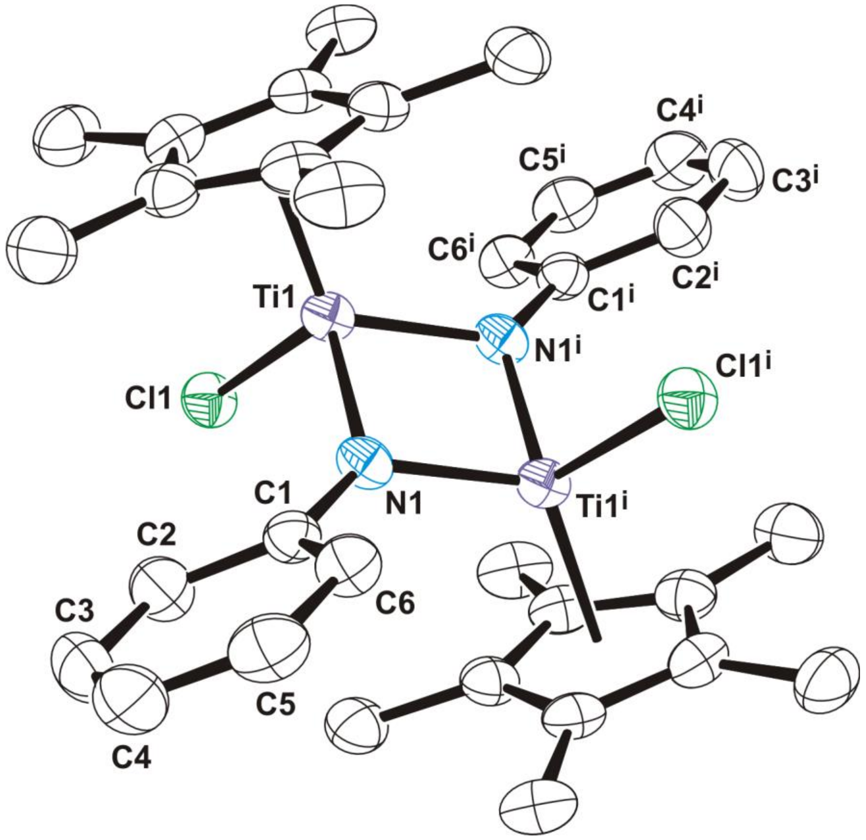

Figure S2. Perspective view of complex **5** (thermal ellipsoids at the 50% probability level). Hydrogen atoms of the η^5-C_5Me_5 and Ph groups are omitted for clarity. Symmetry code: (i) $1 - x$, $1 - y$, $1 - z$.

Table S3. Selected Lengths (Å) and Angles (deg) for **5**.

Ti(1)-Cl(1)	2.314(1)	Ti(1)-N(1)	1.915(3)
Ti(1)-N(1)^i	1.940(3)	Ti(1)···Ti(1)^i	2.835(1)
Ti(1)-Cm(1)^a	2.044		
Cl(1)-Ti(1)-N(1)	105.1(1)	Cl(1)-Ti(1)-N(1)^i	104.3(1)
N(1)-Ti(1)-N(1)^i	85.3(1)	Ti(1)-N(1)-Ti(1)^i	94.7(1)

^{a} Cm = Centroid of the η^5-C_5Me_5 ring. Symmetry code: (i) $1 - x$, $1 - y$, $1 - z$.

Table S4. Selected Lengths (Å) and Angles (deg) for **3**.

Ti(1)-Ti(2)	2.953(1)	Ti(1)-Ti(3)	2.872(1)
Ti(2)-Ti(3)	3.033(1)	Ti(1)-Cl(12)	2.383(1)
Ti(1)-Cl(13)	2.375(1)	Ti(1)-Cl(4)	2.423(1)
Ti(2)-Cl(12)	2.384(1)	Ti(2)-Cl(23)	2.391(1)
Ti(2)-Cl(4)	2.399(1)	Ti(3)-Cl(13)	2.373(1)
Ti(3)-Cl(23)	2.390(1)	Ti(3)-Cl(4)	2.404(1)
Ti(1)-Cm(1)[a]	2.029	Ti(2)-Cm(2)[a]	1.986
Ti(3)-Cm(3)[a]	2.031		
Ti(1)-Cl(12)-Ti(2)	76.6(1)	Ti(1)-Cl(13)-Ti(3)	74.5(1)
Ti(2)-Cl(23)-Ti(3)	78.8(1)	Ti(1)-Cl(4)-Ti(2)	75.5(1)
Ti(1)-Cl(4)-Ti(3)	73.0(1)	Ti(2)-Cl(4)-Ti(3)	78.3(1)
Cl(12)-Ti(1)-Cl(13)	95.7(1)	Cl(12)-Ti(2)-Cl(23)	101.2(1)
Cl(13)-Ti(3)-Cl(23)	97.6(1)	Cl(4)-Ti(1)-Cl(12)	102.4(1)
Cl(4)-Ti(1)-Cl(13)	105.6(1)	Cl(4)-Ti(2)-Cl(12)	103.1(1)
Cl(4)-Ti(2)-Cl(23)	99.1(1)	Cl(4)-Ti(3)-Cl(13)	106.3(1)
Cl(4)-Ti(3)-Cl(23)	99.0(1)		

[a] Cm = Centroid of the η^5-C_5Me_5 ring.

Table S5. Selected Lengths (Å) and Angles (deg) for **6**.

Ti(1)-Ti(1)^i	2.936(1)	Ti(1)-N(1)	2.017(3)
Ti(1)^i-N(2)	2.146(9)	Ti(1)^{ii}-N(2)	2.099(10)
Ti(1)-Cl(1)	2.391(1)	Ti(1)-Cl(1)^i	2.399(1)
N(1)-N(2)	1.099(9)	Ti(1)-Cm(1)[a]	2.040
N(1)-Ti(1)-Cl(1)	95.6(1)	N(1)-Ti(1)-Cl(1)^i	95.4(1)
Cl(1)-Ti(1)-Cl(1)^i	95.9(1)	Ti(1)-Cl(1)-Ti(1)^{ii}	75.6(1)
Ti(1)-N(1)-Ti(1)^i	93.4(2)	Ti(1)-N(1)-N(2)	170.2(7)
N(1)-N(2)-Ti(1)^i	68.3(5)	N(1)-N(2)-Ti(1)^{ii}	70.4(5)
Ti(1)^i-N(2)-Ti(1)^{ii}	87.5(4)	N(1)-Ti(1)^i-N(2)	30.4(3)
N(1)-Ti(1)^{ii}-N(2)	30.9(3)	N(2)-Ti(1)^{ii}-Cl(1)	126.1(3)
N(2)-Ti(1)^i-Cl(1)^i	125.7(3)	N(2)-Ti(1)^i-Cl(1)^{ii}	87.9(3)
N(2)-Ti(1)^{ii}-Cl(1)^{ii}	89.2(3)		

[a] Cm = Centroid of the η^5-C_5Me_5 ring. Symmetry code: (i) $1 - y, x - y, z$; (ii) $1 - x + y, 1 - x, z$.

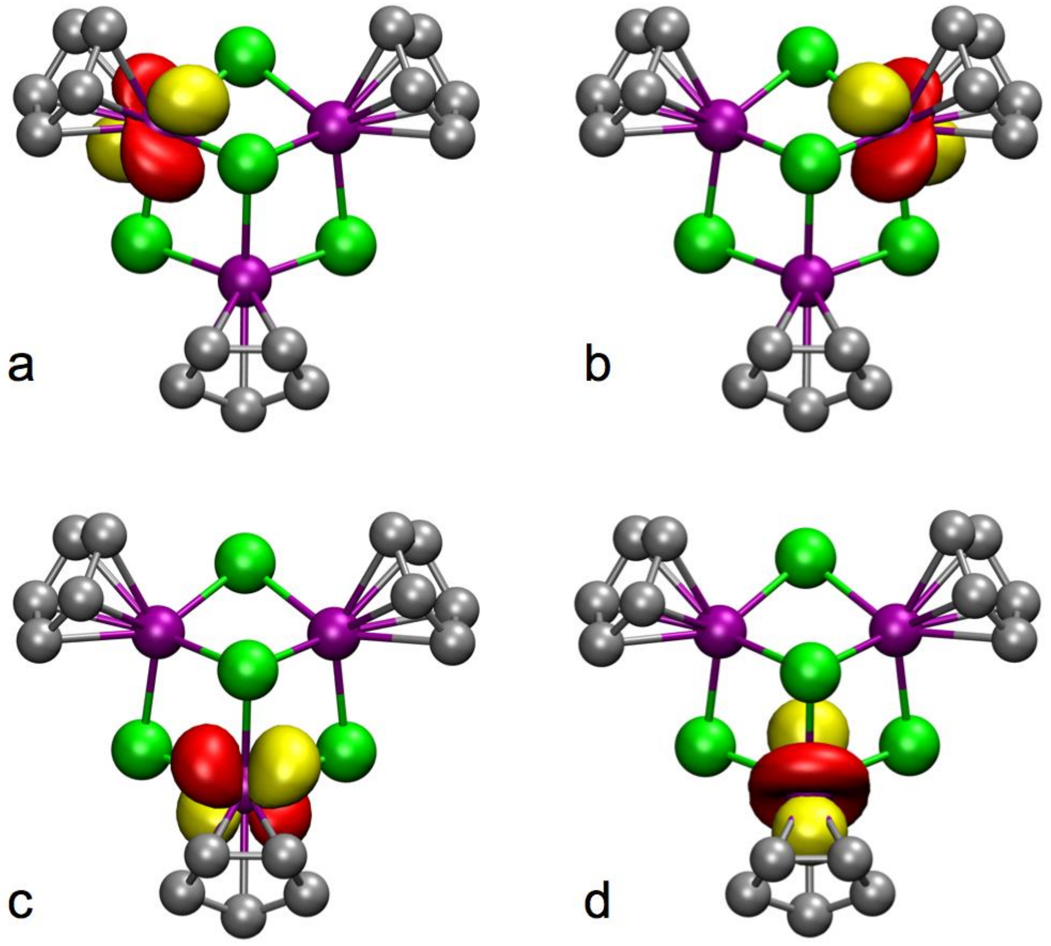

Figure S3. Compound **3** half full alpha orbitals for a) Ti2 and b) Ti3, and half full beta orbitals for c and d) Ti1. Methyl groups of the η^5-C_5Me_5 ligands are omitted for clarity.

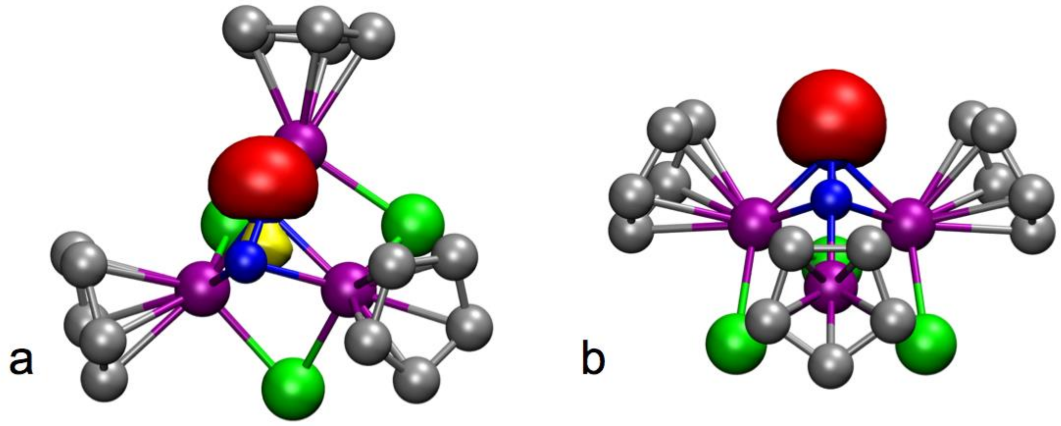

Figure S4. Compound **6** lone pairs on a) N1, and b) N2 within the N_2 unit. Methyl groups of the η^5-C_5Me_5 ligands are omitted for clarity.

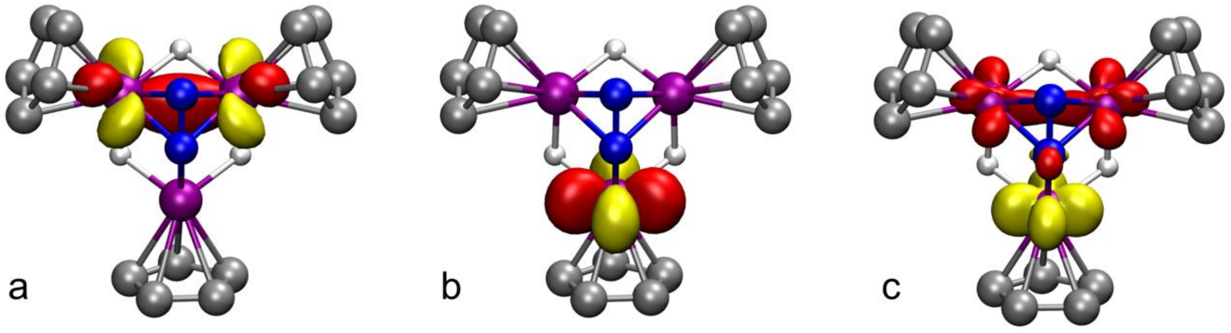

Figure S5. Compound **6'** half full orbitals for a) Ti2–Ti3 (alpha), b)Ti1 (beta), and c) spin density. Methyl groups of the η^5-C_5Me_5 ligands are omitted for clarity.

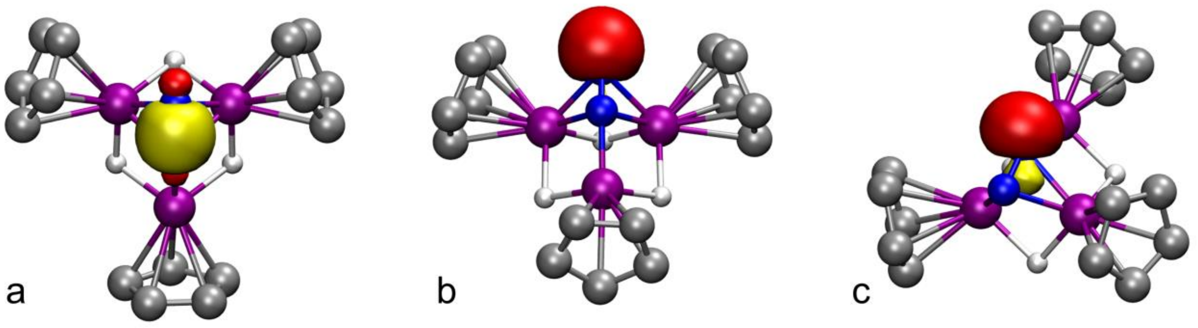

Figure S6. Compound **6'** a) N1–N2 bond, and lone pairs on b) N2, and c) N1. Methyl groups of the η^5-C_5Me_5 ligands are omitted for clarity.

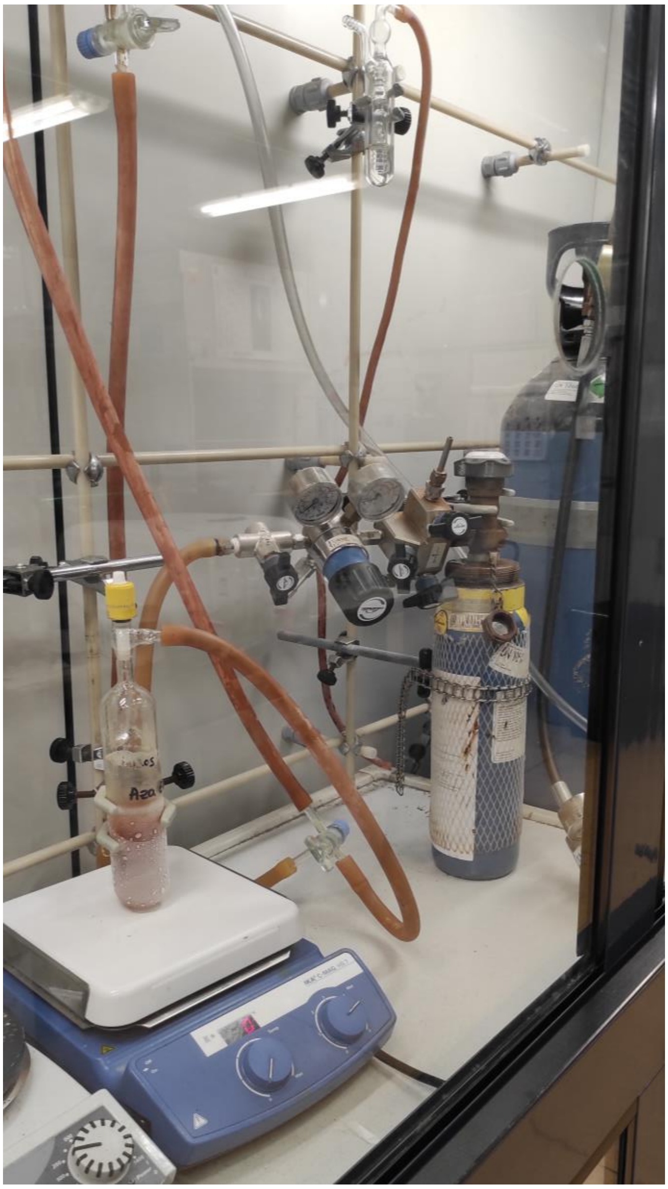

Figure S7. Photograph of the experimental setup for the cyclic preparation of NH_4Cl.

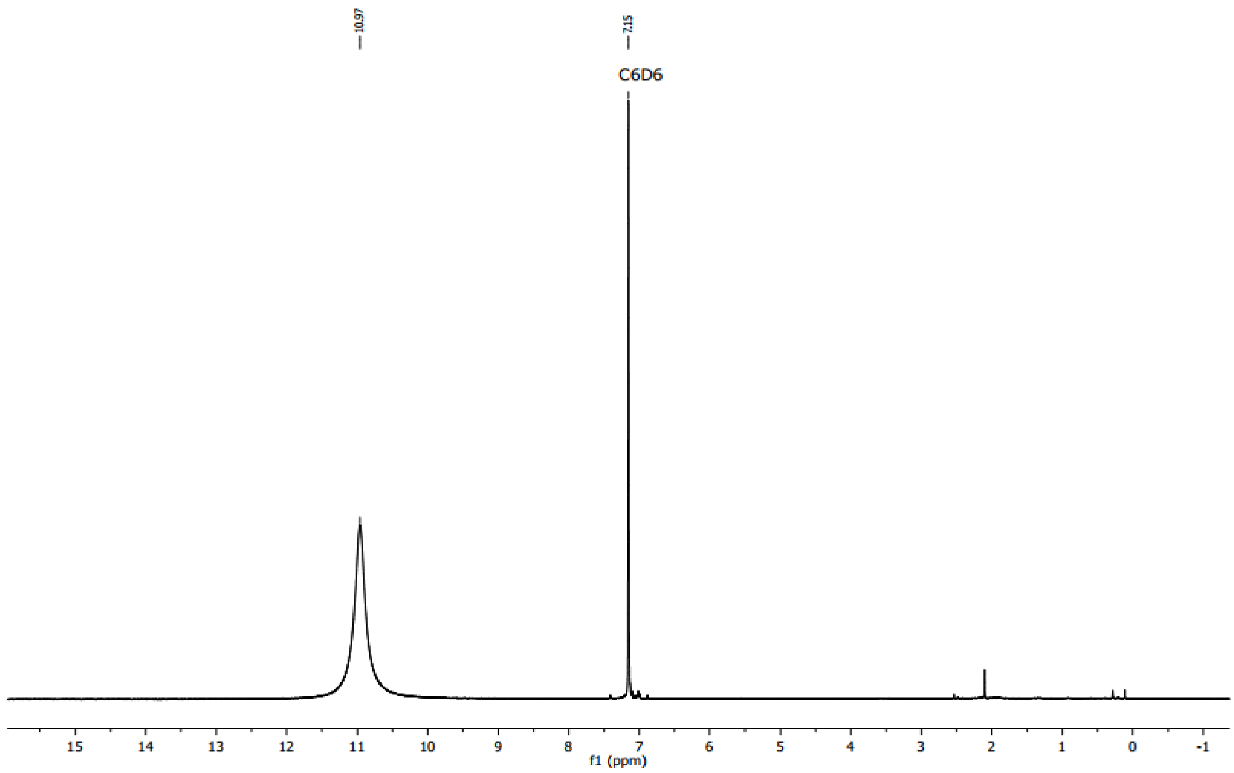

Figure S8. ¹H NMR spectrum (300 MHz, [D$_6$]benzene, 20 °C) of [{TiCp*(μ-Cl)}$_3$(μ$_3$-Cl)] (**3**).

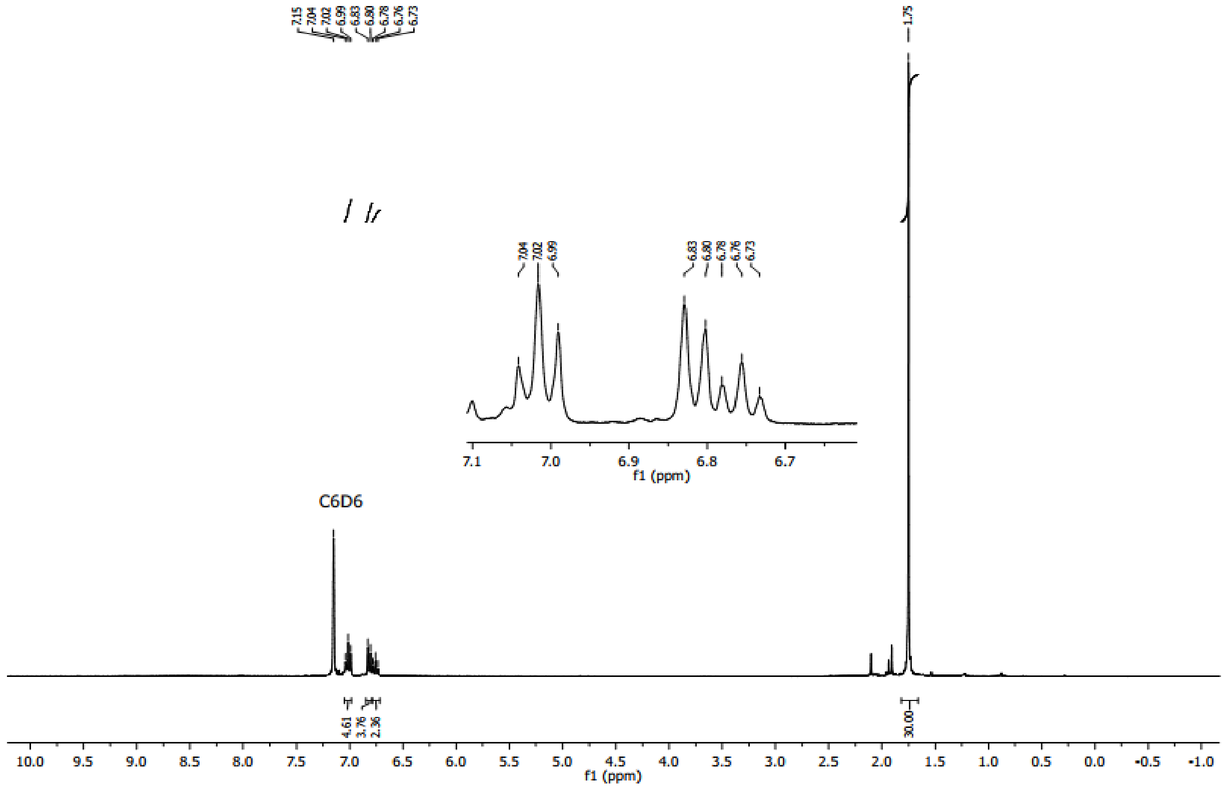

Figure S9. ¹H NMR spectrum (300 MHz, [D$_6$]benzene, 20 °C) of [{TiCp*Cl(μ-NPh)}$_2$] (**5**).

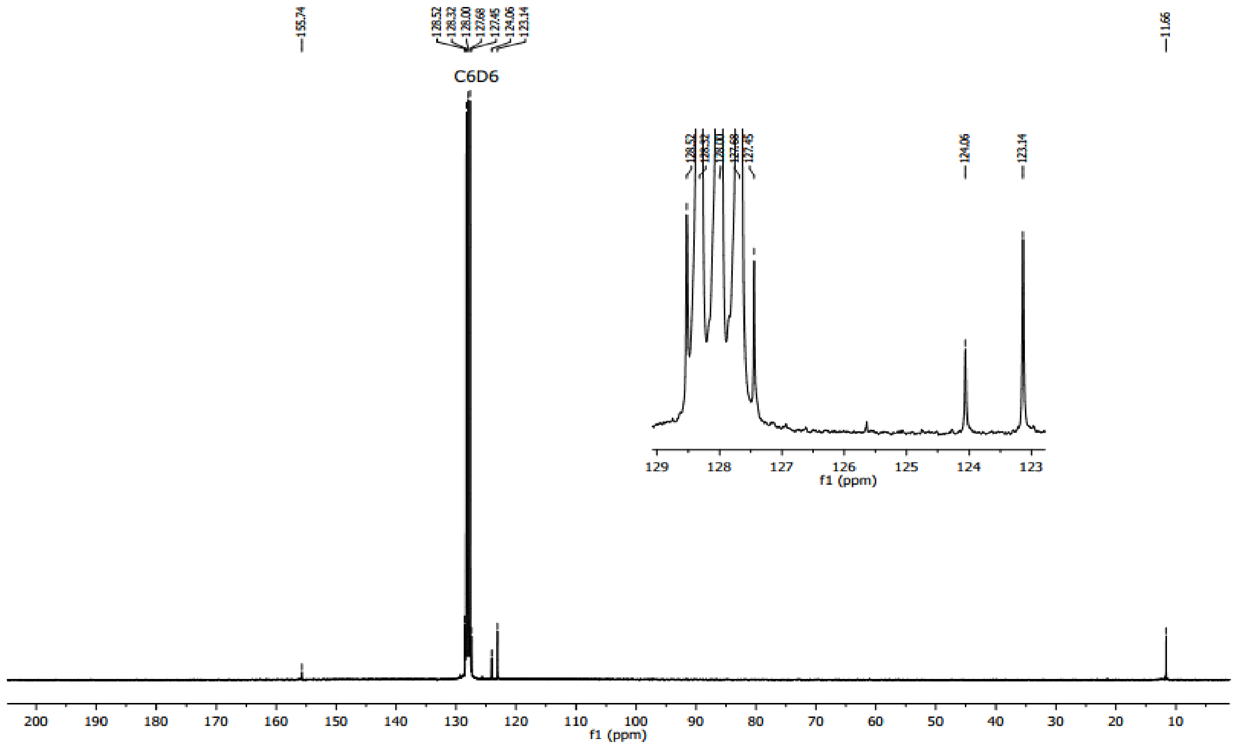

Figure S10. $^{13}C\{^{1}H\}$ NMR spectrum (75 MHz, [D$_6$]benzene, 20 ºC) of [{TiCp*Cl(μ-NPh)}$_2$] (**5**).

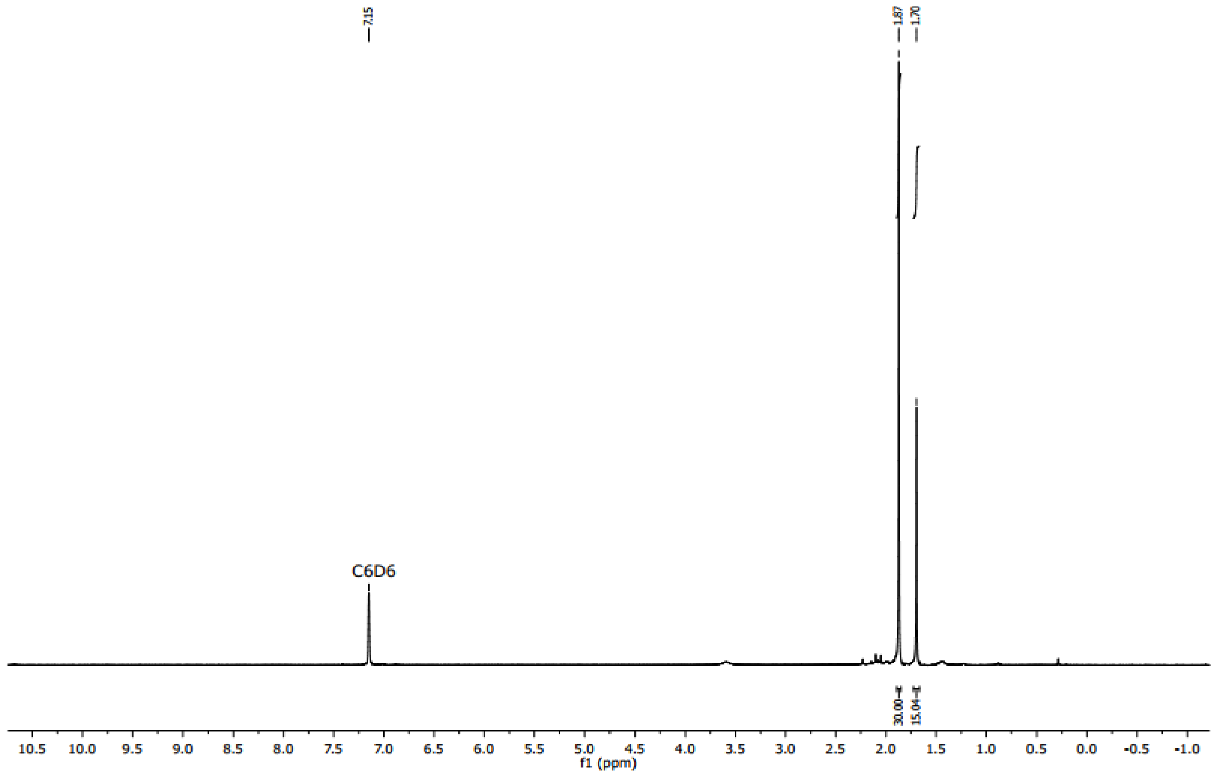

Figure S11. ^{1}H NMR spectrum (300 MHz, [D$_6$]benzene, 20 ºC) of [{TiCp*(μ-Cl)}$_3$(μ$_3$-η^1:η^2:η^2-N$_2$)] (**6**).

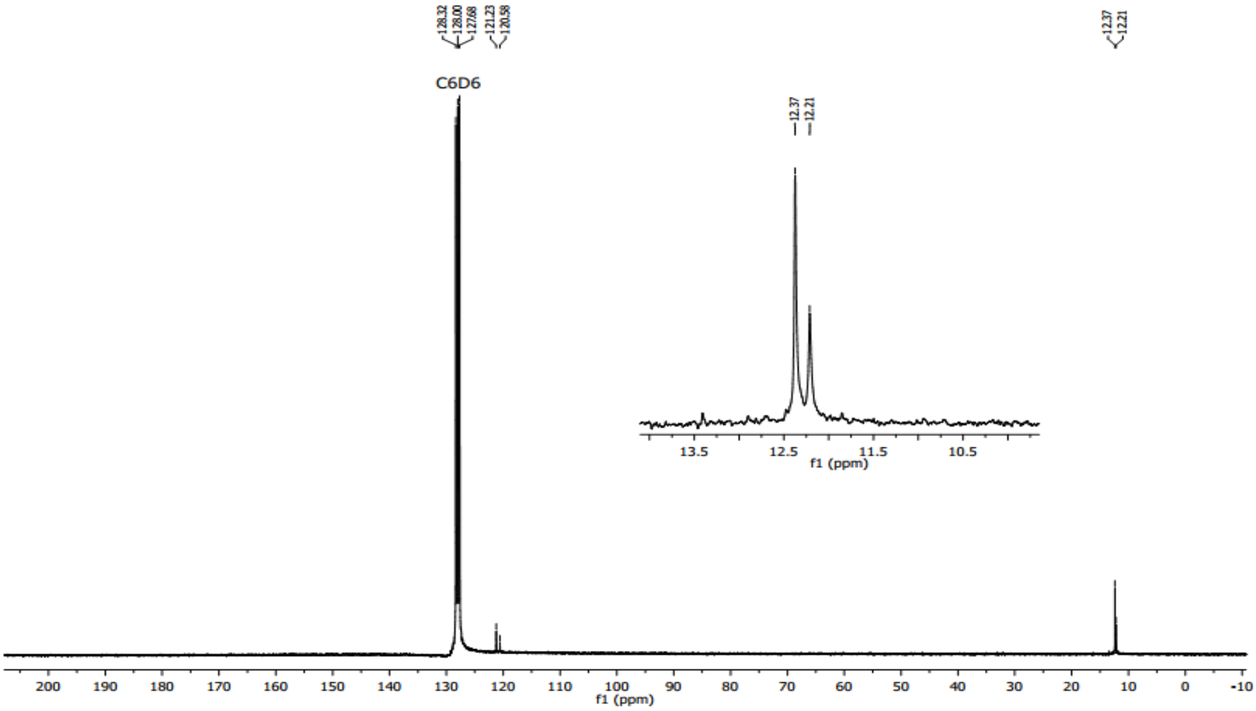

Figure S12. $^{13}C\{^{1}H\}$ NMR spectrum (75 MHz, [D$_6$]benzene, 20 ºC) of [{TiCp*(μ-Cl)}$_3$(μ$_3$-η^1:η^2:η^2-N$_2$)] (**6**).

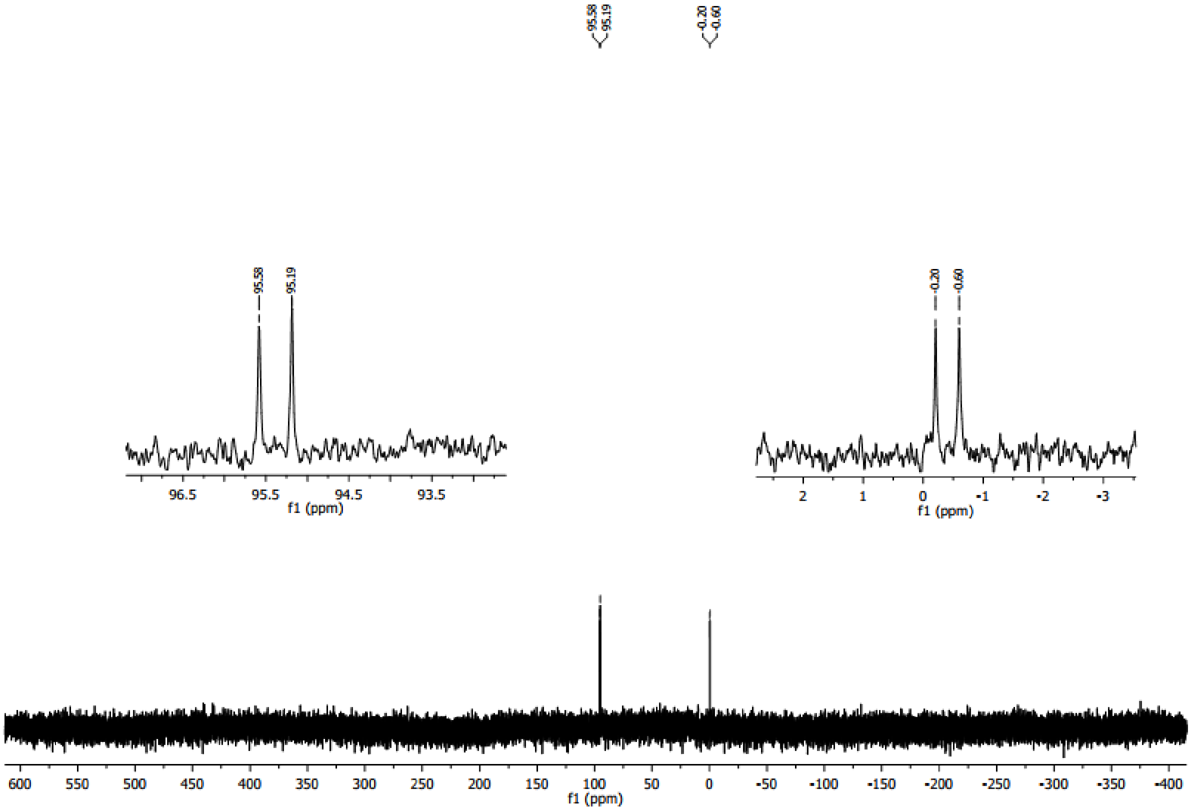

Figure S13. ^{15}N NMR spectrum (50.7 MHz, [D$_6$]benzene, 20 ºC) of [{TiCp*(μ-Cl)}$_3$(μ$_3$-η^1:η^2:η^2-^{15}N$_2$)] (**6-^{15}N$_2$**).

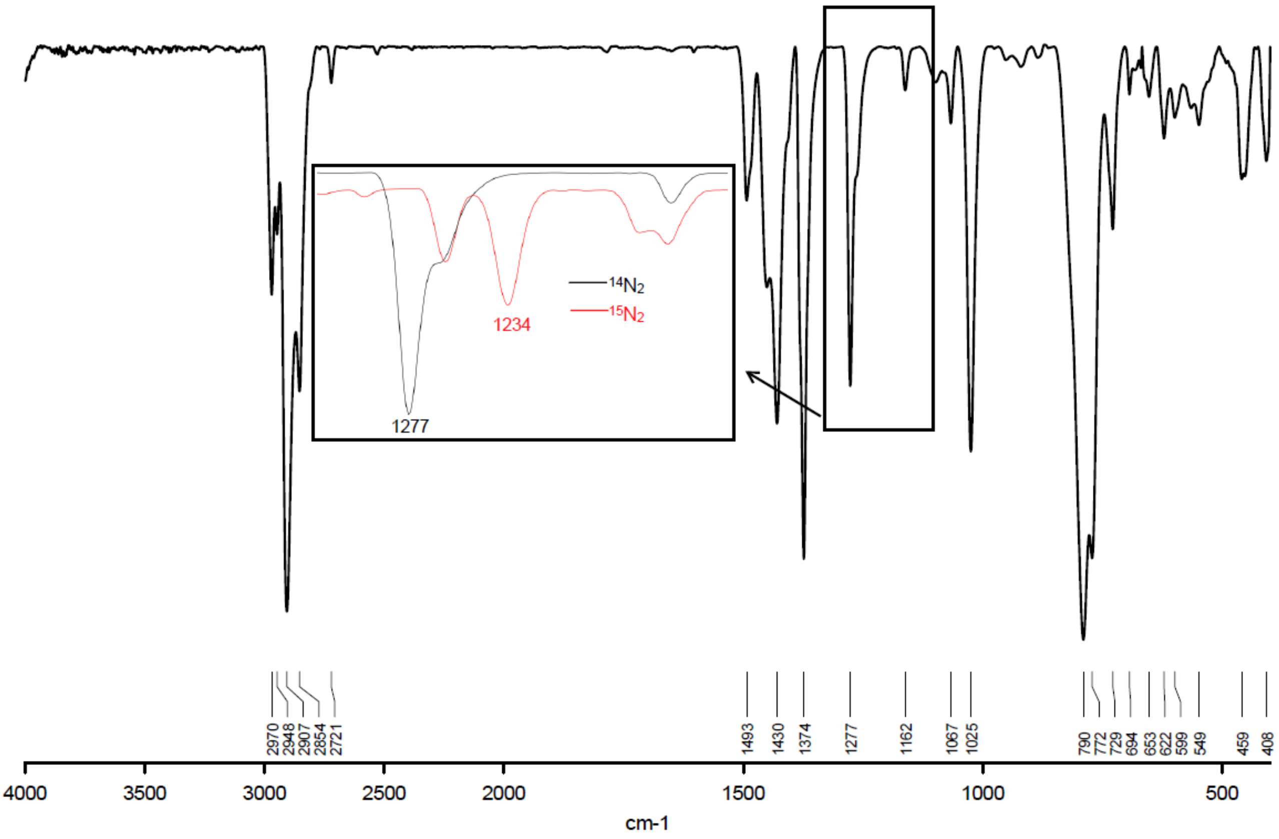

Figure S14. IR spectrum (KBr) of [{TiCp*(μ-Cl)}$_3$(μ$_3$-η^1:η^2:η^2-N$_2$)] (**6**). In the box: ν(N-N) band absorption of **6** (black) and **6-^{15}N$_2$** (red).

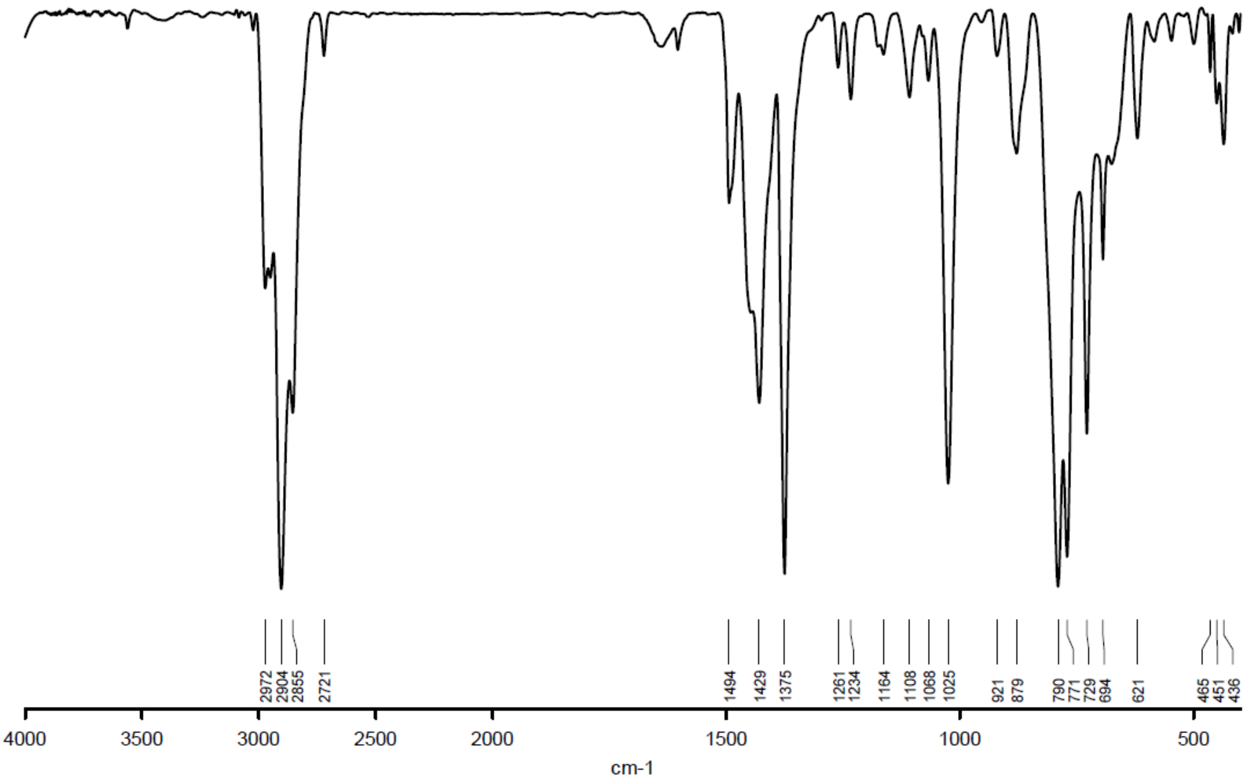

Figure S15. IR spectrum (KBr) of [{TiCp*(μ-Cl)}$_3$(μ$_3$-η^1:η^2:η^2-^{15}N$_2$)] (**6-^{15}N$_2$**).

Cartesian Coordinates (in xyz format)

Complex 3

Ti	-0.50419400	-0.56195200	-1.59087000
Ti	-0.12807900	-0.36212800	1.69425100
Ti	2.50590400	-0.47145300	-0.22956100
Cl	0.64402300	1.19521500	-0.10704500
Cl	-1.53422900	-1.79264400	0.29950900
Cl	1.52898700	-1.94316900	-1.91748700
Cl	2.05103000	-1.44174500	1.97178900
C	-1.89627600	-1.04669600	-3.47645100
C	-0.77452400	-0.30662900	-3.94302100
C	-0.80981200	0.97990200	-3.33507300
C	-1.96572400	1.03778800	-2.49741000
C	-2.63730300	-0.21344800	-2.59298200
C	-2.25939100	-2.44446800	-3.88003800
H	-2.79571900	-2.96370600	-3.08457800
H	-1.37358600	-3.03514400	-4.11762500
H	-2.90326500	-2.44787600	-4.76611700
C	0.23384700	-0.78189700	-4.94524300
H	0.41083700	-1.85403600	-4.85838400
H	1.19572300	-0.28709200	-4.81160800
H	-0.10496300	-0.58062500	-5.96670900
C	0.11213000	2.12987200	-3.60861600
H	0.31706400	2.70154500	-2.70201800
H	-0.31991000	2.81972500	-4.34148100
H	1.06746000	1.78959300	-4.00797400
C	-2.43441500	2.25683700	-1.76104400
H	-3.14362600	1.99980700	-0.97457600
H	-2.93561900	2.95948700	-2.43528500
H	-1.60211200	2.78761000	-1.29544300
C	-3.93034500	-0.57523300	-1.92653100
H	-4.07562000	-0.01360900	-1.00387400
H	-3.96527000	-1.63330100	-1.66629200
H	-4.78116800	-0.36376900	-2.58228900
C	-1.01105600	1.57966700	2.79316400
C	-2.04822700	0.61397300	2.70124100
C	-1.66128300	-0.53045400	3.46008400
C	-0.37746500	-0.26871800	4.01566100
C	0.02955000	1.03338200	3.59141900
C	-1.03655900	2.95408700	2.19817700
H	-0.03229600	3.31539700	1.97905700
H	-1.50442600	3.66330900	2.88784900
H	-1.59854500	2.97706000	1.26483900
C	-3.36203200	0.77965800	2.00133700
H	-4.15110300	1.03881500	2.71424300
H	-3.65931900	-0.13793200	1.49352100
H	-3.32004700	1.57266200	1.25586300
C	-2.50893100	-1.74230000	3.69899800
H	-1.90256100	-2.61542100	3.94123100

H	-3.10206000	-1.98971900	2.81866800
H	-3.19868300	-1.57744300	4.53319300
C	0.37434900	-1.15601100	4.96088200
H	1.45122700	-1.01806700	4.86872300
H	0.16386700	-2.21000000	4.77767100
H	0.09651300	-0.93819200	5.99706200
C	1.30190500	1.71942700	3.98504000
H	2.12856200	1.01189100	4.05553000
H	1.20107000	2.20911900	4.95891200
H	1.58570200	2.48264800	3.26081700
C	4.12512000	0.26986100	-1.82048500
C	4.66376200	-0.82565600	-1.08328000
C	4.78318000	-0.41853800	0.27596000
C	4.30023700	0.92210600	0.37771200
C	3.91005800	1.34681700	-0.92209400
C	3.90081600	0.28555700	-3.30099100
H	3.49940400	-0.66479800	-3.65218800
H	4.83939600	0.47132700	-3.83253000
H	3.19939200	1.06705500	-3.59026300
C	5.10430500	-2.13312100	-1.66737300
H	4.44117000	-2.44947200	-2.47277500
H	5.11140400	-2.92526300	-0.91828300
H	6.11683700	-2.05743700	-2.07708100
C	5.39989300	-1.21586100	1.38504400
H	4.98027700	-0.94627700	2.35366000
H	6.48000400	-1.04368300	1.42796500
H	5.24077300	-2.28581500	1.24899500
C	4.28299700	1.75863600	1.62052400
H	3.48636900	2.50226800	1.58827900
H	5.22910200	2.29390400	1.75082000
H	4.12445300	1.14810500	2.50942100
C	3.42014700	2.71461800	-1.28588800
H	2.86376500	3.17234700	-0.46845200
H	2.75999500	2.68869600	-2.15194500
H	4.26124800	3.37205100	-1.52683100

Complex 6

Ti	-0.96051200	-1.31749200	0.55172400
Ti	-0.93371600	1.50083400	0.22079800
Ti	1.77665400	0.02945200	0.08432100
Cl	1.12826400	1.96374500	1.50074000
Cl	-2.09707000	0.28960100	1.96735700
Cl	1.09443200	-1.51183300	1.90796800
N	0.08145000	-0.05194700	-0.76171300
N	-1.19813700	-0.07483600	-1.05311900
C	-1.46552000	-3.07016800	-0.99841900
C	-0.72568400	-3.65879200	0.05589500
C	-1.48615900	-3.52988700	1.25792600
C	-2.70756100	-2.87940100	0.93520300

C	-2.68964000	-2.58018200	-0.45503500
C	-1.07863400	-2.97570400	-2.44074800
H	-0.04056300	-3.26404800	-2.59886300
H	-1.20115000	-1.95258600	-2.80071600
H	-1.70488700	-3.62932600	-3.05502200
C	0.58710800	-4.36879300	-0.05540900
H	1.10360700	-4.10932500	-0.97806400
H	0.43692600	-5.45280300	-0.05310500
H	1.24597600	-4.11963600	0.77541200
C	-1.10487400	-4.08671500	2.59481500
H	-1.54233000	-3.50956100	3.40998000
H	-0.02431800	-4.07992500	2.73334600
H	-1.45071900	-5.12040300	2.69810000
C	-3.84421600	-2.62442700	1.87709800
H	-4.53305100	-3.47482100	1.88624300
H	-4.41095700	-1.73899600	1.59043700
H	-3.49323300	-2.46965800	2.89723300
C	-3.78299000	-1.94170900	-1.24878400
H	-4.42408600	-2.69899000	-1.71130800
H	-3.36647400	-1.31970300	-2.03939400
H	-4.41498000	-1.30772100	-0.62676300
C	3.24859000	0.53116600	-1.70642000
C	3.80771200	1.09843900	-0.53185000
C	4.11797300	0.03140000	0.36325400
C	3.77877000	-1.19773700	-0.27763900
C	3.23051100	-0.88737200	-1.54930600
C	2.80169000	1.25472900	-2.94029700
H	2.66865500	2.31941200	-2.75389100
H	1.85381600	0.86147500	-3.31046500
H	3.53941900	1.15166100	-3.74248400
C	4.09556400	2.54739700	-0.28457900
H	3.49317500	3.19150000	-0.92328700
H	5.14848100	2.77150800	-0.48204100
H	3.88304100	2.82615600	0.74770900
C	4.75661300	0.17208600	1.71284300
H	4.45993300	-0.63805800	2.38000000
H	4.47324300	1.10999000	2.19196300
H	5.84913100	0.15573700	1.63811200
C	4.03485900	-2.56361700	0.28140500
H	5.08875700	-2.83584300	0.16756700
H	3.43965500	-3.32274400	-0.22372800
H	3.79029800	-2.61345600	1.34274200
C	2.75894600	-1.85159900	-2.59517800
H	3.49413500	-1.94521500	-3.40082400
H	1.81872900	-1.52469500	-3.04165600
H	2.60025200	-2.84596700	-2.18030200
C	-1.39609100	2.86043400	-1.69394300
C	-2.63298500	2.52736100	-1.06706700
C	-2.65561200	3.13612700	0.21784400
C	-1.42490100	3.82375900	0.39743200

C	-0.65396300	3.66339600	-0.79392400
C	-1.00104500	2.43251900	-3.07235000
H	-1.12792800	1.35437000	-3.18686300
H	0.03945200	2.67230100	-3.28556400
H	-1.61993300	2.92859600	-3.82572400
C	-3.73203200	1.74355100	-1.70787900
H	-3.32094000	0.95003400	-2.32982500
H	-4.35351600	2.38619800	-2.33968800
H	-4.38207400	1.27984500	-0.96590300
C	-3.80410400	3.12148800	1.17960500
H	-3.46427500	3.19836200	2.21225400
H	-4.38500100	2.20335800	1.09591700
H	-4.47710600	3.96251400	0.98599300
C	-1.04303000	4.66406000	1.57674600
H	-1.36700800	5.70051400	1.43674400
H	0.03591400	4.66832500	1.72755900
H	-1.50009300	4.29703700	2.49616100
C	0.67095600	4.31016100	-1.05113200
H	0.53743700	5.36572400	-1.30732900
H	1.19338500	3.83338100	-1.87884600
H	1.31695200	4.25652900	-0.17579800

Complex 6´

Ti	-1.00132900	-1.28228800	0.18023200
Ti	-0.94808000	1.37422000	-0.09595900
Ti	1.49319400	-0.04119000	-0.29888600
H	0.77489600	1.45062800	0.55344800
H	-1.64736000	0.17634400	1.16460700
H	0.71862300	-1.29219200	0.83749400
N	-0.12662600	-0.10918100	-1.29930400
N	-1.45770900	-0.09413700	-1.38399100
C	-1.79630100	-3.12637400	-1.16431200
C	-0.77229200	-3.61975400	-0.31826700
C	-1.20759900	-3.46541400	1.03289000
C	-2.51044100	-2.88542700	1.01230300
C	-2.86723000	-2.66602900	-0.34726200
C	-1.78304400	-3.06563100	-2.65990000
H	-0.80919600	-3.34068100	-3.06418800
H	-2.01417600	-2.05505400	-3.00267400
H	-2.52519300	-3.74809200	-3.08489100
C	0.50908400	-4.25703100	-0.76209000
H	0.88497300	-3.80307500	-1.67926700
H	0.37161600	-5.32637100	-0.95342400
H	1.28323500	-4.15440400	-0.00357700
C	-0.46627000	-3.91871200	2.25560700
H	-0.68665700	-3.28710300	3.11775300
H	0.61213700	-3.89112900	2.09954700
H	-0.73852600	-4.94457000	2.52529200
C	-3.36990500	-2.61765500	2.21331600

H	-3.94793900	-3.50664400	2.48592100
H	-4.07575200	-1.80786200	2.02851300
H	-2.77318300	-2.33831500	3.08288000
C	-4.13077400	-2.05804000	-0.87129000
H	-4.79358400	-2.82127000	-1.29130100
H	-3.90739800	-1.33714400	-1.65979900
H	-4.68173600	-1.53535100	-0.08928500
C	3.59263600	0.42345800	-1.29558000
C	3.55737400	1.10782500	-0.04842300
C	3.48446300	0.13036800	0.98674800
C	3.50335000	-1.15715000	0.37883800
C	3.56050900	-0.97773800	-1.03041500
C	3.70089800	1.05318400	-2.65351300
H	3.27018700	2.05492300	-2.66715200
H	3.18332300	0.46414800	-3.41194300
H	4.74699500	1.14160000	-2.96433500
C	3.69234400	2.58472400	0.16794600
H	3.48398900	3.14410100	-0.74260200
H	4.70989600	2.83709100	0.48324900
H	3.00816200	2.94018700	0.93934900
C	3.48918100	0.40895000	2.45971700
H	2.96330700	-0.36952600	3.01355900
H	3.00196900	1.35798900	2.68557000
H	4.51145600	0.45978900	2.85059200
C	3.54874100	-2.45877100	1.12075000
H	4.55353500	-2.64476700	1.51331000
H	3.28947400	-3.29764600	0.47666400
H	2.85648800	-2.46278200	1.96324900
C	3.62532600	-2.05964200	-2.06805300
H	4.65854200	-2.24286000	-2.38017700
H	3.05783400	-1.79496900	-2.96204200
H	3.22414200	-3.00039500	-1.69249400
C	-1.66212600	2.92895800	-1.80143300
C	-2.75651900	2.68396600	-0.92471700
C	-2.40265300	3.16490900	0.36638500
C	-1.07923600	3.69205500	0.28796000
C	-0.62824200	3.55138900	-1.05943800
C	-1.63424800	2.56275800	-3.25244400
H	-1.91455900	1.51625700	-3.38749700
H	-0.64166400	2.70038700	-3.68087100
H	-2.33334300	3.17976700	-3.82503400
C	-4.03839100	2.02751800	-1.33246500
H	-3.83703800	1.15922100	-1.96228900
H	-4.67309100	2.71544900	-1.90003500
H	-4.60960500	1.68627900	-0.46896000
C	-3.28146800	3.18074700	1.58302700
H	-2.70339200	3.06282900	2.50071300
H	-4.01771400	2.37743900	1.55592200
H	-3.82562500	4.12776100	1.65938400
C	-0.33439100	4.35910900	1.40620800

H	-0.58245000	5.42396200	1.46698800
H	0.74400100	4.27630200	1.27128500
H	-0.57591300	3.91395100	2.37282400
C	0.67789100	4.03680200	-1.61085200
H	0.55786600	5.00278300	-2.11195000
H	1.09047600	3.33678900	-2.33889900
H	1.41676500	4.16306800	-0.82228000
